# Comparative genomics reveals distinct diversification patterns among LysR-type transcriptional regulators in the ESKAPE pathogen *Pseudomonas aeruginosa*

**DOI:** 10.1099/mgen.0.001205

**Published:** 2024-02-29

**Authors:** Jamie Deery, Muireann Carmody, Rhiannon Flavin, Malwina Tomanek, Maria O'Keeffe, Gerard P. McGlacken, F. Jerry Reen

**Affiliations:** ^1^​ School of Microbiology, University College Cork, Cork, Ireland; ^2^​ School of Chemistry, University College Cork, Cork, Ireland; ^3^​ Synthesis and Solid State Pharmaceutical Centre, University College Cork, Cork, Ireland

**Keywords:** LysR-type transcriptional regulator (LTTR), *Pseudomonas aeruginosa*, evolution, oxidative stress, antimicrobial resistance, PqsR (MvfR)

## Abstract

*Pseudomonas aeruginosa*, a harmful nosocomial pathogen associated with cystic fibrosis and burn wounds, encodes for a large number of LysR-type transcriptional regulator proteins. To understand how and why LTTR proteins evolved with such frequency and to establish whether any relationships exist within the distribution we set out to identify the patterns underpinning LTTR distribution in *P. aeruginosa* and to uncover cluster-based relationships within the pangenome. Comparative genomic studies revealed that in the JGI IMG database alone ~86 000 LTTRs are present across the sequenced genomes (*n*=699). They are widely distributed across the species, with core LTTRs present in >93 % of the genomes and accessory LTTRs present in <7 %. Analysis showed that subsets of core LTTRs can be classified as either variable (typically specific to *P. aeruginosa*) or conserved (and found to be distributed in other *Pseudomonas* species). Extending the analysis to the more extensive Pseudomonas database, PA14 rooted analysis confirmed the diversification patterns and revealed PqsR, the receptor for the Pseudomonas quinolone signal (PQS) and 2-heptyl-4-quinolone (HHQ) quorum-sensing signals, to be amongst the most variable in the dataset. Successful complementation of the PAO1 *pqsR* ^-^ mutant using representative variant *pqsR* sequences suggests a degree of structural promiscuity within the most variable of LTTRs, several of which play a prominent role in signalling and communication. These findings provide a new insight into the diversification of LTTR proteins within the *P. aeruginosa* species and suggests a functional significance to the cluster, conservation and distribution patterns identified.

## Data Summary

The authors confirm all supporting data, code and protocols have been provided within the article or through supplementary data files. All sequence data generated in this work are available in the GenBank database under access numbers OR502010–OR502029. Our raw data, analysis pipelines, codes and associated information are publicly accessible through GitHub (https://github.com/JLouDee/LysR-paper).

**Impact Statement**

Transcriptional regulation is a mechanism through which living cells control gene expression to ensure the appropriate response to external and internal stimuli. Amongst the large repertoire of transcriptional regulator proteins encoded in bacteria, LysR-type tanscriptional regulators (LTTRs) are one of the most abundant families, controlling antimicrobial resistance, cell–cell communication, xenobiotic biodegradation, the oxidative stress response and metabolism. While it has been known since the advent of genome sequencing that bacteria can encode a diverse repertoire of LTTR proteins, little is known about the relationship of these proteins and how their distribution can be mapped to better understand their functional roles. Comparative analysis of available genomic datasets of the opportunistic ESKAPE pathogen *Pseudomonas aeruginosa* revealed that LTTR homologues were distributed in either less than 7 % or more than 93 % of genomes. The most highly conserved LTTRs were species-wide with a prominent role in the oxidative stress response. The most variable LTTRs were typically species specific, and generally uncharacterized, though proteins with a key role in signalling and communication were notably present. Exploring the functional significance of evolutionary divergence in PqsR, a LTTR protein controlling the alkyl quinolone (AQ) quorum-sensing signalling system, pyocyanin (PYO) and 2-heptyl-4-quinolone (HHQ) production was restored in a PAO1 pqsR-mutant following complementation with seven variant *P. aeruginosa* cluster representatives. Together, these findings provide a new insight into the diversification of LTTR proteins within the *P. aeruginosa* species and suggest a form of sequence flexibility in the variable LTTRs identified in this study.

## Introduction

Gene regulation is a vital process for the survival of micro-organisms as it allows them to manage their available resources, adapt to new environmental niches and mediate an efficient response to stress [1]. In adapting to these diverse conditions, the micro-organism requires a system, which allows for the coordination of transcriptional, post-transcriptional, translational and epigenetic regulation. These interconnected processes require the concerted action of interwoven yet discrete signal transduction networks to ensure they are carried out efficiently [1].

The LysR family is one of the largest known families of transcriptional regulators in prokaryotes and they are highly relevant in the study of pathogenesis and infection [2]. Along with the TetR family of transcriptional regulator, these accounted for 28.4 % of bacterial and archaeal proteins encoded in 761 non-redundant genomes evaluated by Perez-Rueda and co-workers in 2018 [3]. Henikoff and colleagues first classified LTTRs as a distinct family in 1988 [4]. The study found nine distinct LTTRs but this number has grown since, with 767 unique LTTRs identified in the *Pseudomonas* genus alone in 2013, across 40 available genomes [5]. Six families of transcriptional regulator predominate in *P. aeruginosa*: LysR, TetR, AraC, LuxR, OmpR and GntR family proteins are represented by 269/371 of the identified transcription factors in the PAO1 genome [6]. Of these, approximately 120 are LTTR family proteins, and the frequency with which *P. aeruginosa* encodes LTTR proteins is amongst the highest in the microbial domain [5], perhaps a reflection of its niche-adaptability and resilience. LTTRs have the ability to regulate the expression of numerous genes with varied functions, while still maintaining their conserved modular structure. The diverse range of functions identified in *P. aeruginosa* includes virulence, response to oxidative stress, metabolism, toxin production, cell division, motility, quorum sensing (QS) and nitrogen fixation [2]. Proteins such as PqsR [also referred to as MvfR (multiple virulence factor regulator)], AmpR, MexT, OxyR and BvlR have been shown to influence a range of adaptive processes in *P. aeruginosa* [7–11]. Mutations in some of these proteins have previously been identified during infection in patients with CF [12, 13]. In some cases this was contingent on linkages between upstream and downstream regulators, suggesting remodelling of regulatory networks might be important [13]. The adaptive mutations are interesting insofar as transcriptional regulator families evolve quickly as they respond to signals that they meet in a new environment [14]. In order to adapt to new environments, it is thought that bacteria acquire small changes through their transcriptional regulatory networks until an optimum arrangement is met [15]. For the most part, the nature of these signals and the basis of their selective pressure remains to be determined. In the case of PqsR very little is known about the diversification of the virulence regulator even though it is responsible for regulating the expression of 18 % of the *P. aeruginosa* genome [16]. PqsR is a key component of the alkylquinolone QS system, linked to the production of exotoxins, exoenzymes, secondary metabolites (e.g. pyocyanin, hydrogen cyanide and rhamnolipids), lectin as well as biofilm development [17]. With PqsR, as well as AmpR, being considered as potential drug targets [7, 18], it is important that we have a greater understanding of these proteins.

LTTRs are thought to have arisen in bacteria, with strong evidence suggesting that they can be acquired through horizontal transfer [2]. However, in contrast to the predominantly AT-rich bias of DNA acquisition, genes encoding LTTRs have high GC content due to the Lys/Arg ratio common to the proteins [4, 19]. While several of these proteins have been studied with considerable interest owing to their role in co-ordinating the cell response to external challenges, changing environmental conditions and the host–pathogen symbiotic relationship, very little is understood about how or why such a large repertoire emerged and is maintained in *P. aeruginosa*. This represents a significant gap in knowledge where it relates to a signal-responsive regulatory system with a key role in bacterial adaptation and ecosystem colonization. Therefore, we undertook a census of LTTR-encoding genes from genome sequences available through the IMG database and uncovered interesting patterns of distribution and sequence constraint. An extension of this analysis into the larger Pseudomonas database (www.pseudomonas.com), DIAMOND BLASTP was undertaken using the PA14 LTTR repertoire as a root. This confirmed the diversification patterns identified in the IMG analysis, with a distinct gradient from highly conserved to highly variable proteins evident. Functional complementation of one of the variable LTTRs, PqsR, suggests a degree of sequence freedom or flexibility within less conserved LTTR proteins, where representative sequences for each variant tested restored key phenotypes. These data suggest that microbial pathogens such as *P. aeruginosa* encode a complex network of LTTR encoding proteins with distinct conservation profiles to co-ordinate a refined response to environmental challenges.

## Methods

### Recovery of *P. aeruginosa* LTTR protein sequences from IMG database

LTTR protein sequences were retrieved from all of the 699 available permanent, draft and finished *P. aeruginosa* genomes in the integrated microbial genomes (IMGs) [20]. The following protein conserved domain families with accession numbers COG0583 (LTTR conserved sequence), Pfam00126 (LTTR DNA binding domain) and Pfam03466 (LTTR substrate binding domain) were applied to identify ~260 k LTTR homologues. These LTTR amino acid sequences were then filtered by removing duplicate sequences and sequences less than 150 amino acids and larger than 350 amino acids using the Biolinux command line [21]. The LTTR proteins (~86 000) were clustered into groups using the CD-Hit programme v4.6 (+OpenMP) [22, 23], which uses a greedy clustering algorithm to group sequences together based on their length and similarity. A sequence identity threshold of 95 % was used to cluster the sequences into groups with one representative LTTR per group. A high threshold (greater than the 80 % threshold previously used to cluster LTTRs in *P. aeruginosa* [5]) was used initially in order to ensure that each group represented a close homology set and that divergence of homologous LTTR proteins could be captured [24]. Therefore, each of these groups represents a unique LTTR and its closest homologues.

### Construction of representative LTTR tree

A sequence alignment was carried out on the representative LTTR protein sequences using the default parameters of the multiple alignment tool muscle v3.8.31 [25]. The muscle output was then used to create a cluster tree. A maximum-likelihood tree [26] was built with PhyML v3.0.1 [27] using the WAG amino acid substitution model of evolution [28] and four categories of substitution rates. Branch supports were evaluated using the approximate likelihood-ratio test (aLRT) [29]. Phyml outputs a Newick file, which was visualized and exported using the web-based tool Interactive Tree Of Life (iTOL v4.4.2) [30]. Cluster designations were assigned based on the position of nodes in the assembled tree, as previously reported [5].

### Analysis of the distribution of LTTRs across the *P. aeruginosa* pangenome

A CD-Hit of 80 % was performed using the same parameters to ensure all of the LTTRs were captured by the correct representative sequence. As the aim here was to ensure that all homologous of a particular LTTR were captured in the same group, the threshold for inclusion was relaxed to 80 %. The distribution of the LTTRs was analyzed across the genomes using RStudio (v1.1a) [31, 32] to manipulate the data from the CD-Hit results using the dplyr package v0.8.3 [33] and to create a histogram and scatter plot using the ggplot2 package v3.2.0 [34].

To investigate if the distribution of LTTRs across the genomes follows a syntenic pattern, Diamond blast analysis was performed for each of the PAO1 and PA14 LTTR repertoires using the www.pseudomonas.com platform against the following *P. aeruginosa* genomes: 2192, DK2, PA96, PA1R, PAK, PA7, M18, LESB18. Gene Ontology (GO) analysis was then undertaken using the GO function of the Pseudomonas database. The frequency of GO terms in each region tested was determined using the ‘remove duplicates’ and ‘COUNTIF’ functions in Excel.

### Comparative genomic analysis of the LTTR diversification

A CD-Hit using a sequence identity threshold of 100 % using the same parameters as above was then used to analyze the variation of LTTRs across the genomes. The variation of each LTTR was calculated by dividing the number of clusters by the number of genomes the protein is present in. This number was then multiplied by 100 to give a unit of variance. The calculations were carried out in R and the top 10 most and least variable LTTR results were tabulated and formatted using the tidyverse package v1.3.0 [35] and pie-charts were created using ggplot2 [34].

### McDonald–Kreitman analysis of selective pressure

The standard McDonald–Kreitman test [36] was performed on full-length LTTR nucleotide sequences of the most variable LTTRS in *P. aeruginosa*. The neutrality index (NI) was calculated based on the ratio of polymorphisms to substitutions as follows: NI = (Pn/Ps)/(Dn/Ds), where P signifies polymorphism within the species and D represents divergence between species. The sequences were obtained from the JGI IMG database, and the outliers used were *P. fluorescens* NCTC10783 and *Pseudomonas sp*. AK6U. The *rpoD* housekeeping gene was selected as a control for this analysis [37], with sequences obtained using the Pseudomonas database [38] and blastn [39].

### Data collection and curation for PA14 rooted analysis

A rooted analysis of all known alleles of the *P. aeruginosa* PA14 LTTR genes was performed using the DIAMOND blastp function on the Pseudomonas Database (www.pseudomonas.com) [38, 40]. Sequences with less than 100 % matches for each allele were considered variants. A dataset was then created containing all the relevant LTTR gene IDs, detailing the number of variants per gene, the amino acid sequence for each variant and the number of homologues per variant. Any known biological functions associated with the isolates encoding each gene ID was also captured here. The dataset represented 123 LTTR genes from *P. aeruginosa* PA14, encoding 7748 variants and a total of 265 086 homologues across the *P. aeruginosa* pangenome.

A number of variant sequences present in the database were found to have undefined characters (‘X’). While some sequences presented with only one undefined character, the issue was far more prevalent in others. Therefore, any sequence with an undefined character was removed from the database. Removal of these sequences was implemented using RStudio (v1.1a) and R (v.3.5.3) statistical software programmes with accompanying packages: dplyr, readxl, rowr, seqRFLP, tidyr and xlsx.

### Rooted multiple sequence alignment and cluster trees

The PA14 rooted LTTR database was split into groups based on gene ID (PA14_XXXXX) using the previously specified R programmes. Each group was converted to fasta file format online (http://sequenceconversion.bugaco.com). The alignment was built using mega7 (v.7.0.26) muscle Alignment (all default parameters).

Each group alignment was used to build maximum-likelihood trees using mega7. All default parameters were implemented with the exception of: WAG model of substitution, Gamma distributed with invariant sites (G+I) rates among sites and gaps/missing data treatment – partial deletion at default of 95 %. Trees were visualized in FigTree (v.1.4.3) using default settings.

### Construction of the PqsR cluster tree with metadata

The available host and isolation information was obtained from the JGI IMG database for the 699 genomes. Data manipulation was performed using the tidyverse package in R. LTTR sequences from the 100 % CD-Hit output were aligned using muscle with the default parameters. The tree was created using the Phyml software with the parameters outlined above and the trees were visualized using the web interface iTOL. The metadata scripts were manually created using the available iTOL templates as examples and uploaded through the web interface.

### Functional complementation analysis using *pqsR* sequence variants

*P. aeruginosa* strains were obtained from clinical, environmental and bovine rumen isolates and grown in Lysogeny (LB) broth. Oligonucleotide primers MMvfRF1 and MMvfRR1 were designed to amplify the *pqsR* gene based on the PAO1 reference genome (RefSeq Accession Number: NP_249694.1). The DNA fragment was amplified from the strains using Q5 Hot Start High-Fidelity DNA Polymerase (New England Biolabs) and High GC Enhancer with an annealing temperature of 50 °C. The products were then purified using the QIAGEN QIAquick PCR Purification kit and sent to Eurofins Genomics for sequencing. The protein sequences encoded in the amplicons (GenBank Accession Numbers: OR502010–OR502029) were added to the PqsR sequences previously obtained from the IMG database. A CD-Hit at 100 % was then carried out including these new sequences to identify representative sequences for complementation.

The MMvfRF1 and MMvfRR1 primer sequences include restriction sites compatible with the pBBR1-MCS5 plasmid. Amplicons generated from the representative variant genomes were purified using a QIAGEN PCR purification kit and subsequently restricted using *Eco*R1 and *Xba*I (SuRE/Cut, Roche). pBBR1-MCS5 plasmid was similarly digested to generate sticky ends for cloning of the insert. Ligation proceeded overnight at 16 °C followed by subsequent transformation into chemically competent *Escherichia coli* DH5α cells. Successful constructs were identified by PCR using the gene-specific primers and were conjugated into *P. aeruginosa* PAO1 *pqsR*- [41] by triparental mating with the *E. coli* pRK2013 helper strain. Successful transconjugation was confirmed by antibiotic selection and PCR amplification following plasmid extraction.

### PQS/HHQ extraction and spectrophotometric profiling

*P. aeruginosa* was grown overnight in LB media and transferred to 25 ml fresh LB medium at OD_600 nm_ 0.05 to initiate growth. The culture was left shaking (180 rpm) at 37 °C for 8 h, after which 5 ml of each inoculation was centrifuged at 4000 rpm for 10 min. The supernatant was then passed through a 0.2 µm filter. PQS/HHQ was extracted using acidified ethyl acetate as previously described by Fletcher and colleagues [42]. Ethyl acetate was removed by rotatory evaporation, and residue was recovered in 1 ml methanol. PQS/HHQ extracts were visualized *via* thin-layer chromatography on a silica plate pre-treated with 5 % (wt/vol) KH_2_PO4 for 30 min before activating at 100 °C for 1 h in a hybridization oven. The absorbance at λ_max_ of HHQ was recorded from each extract in cuvettes in the 200–800 nm spectrum using a spectrophotometer using standardized 1 mM solutions of PQS and HHQ as a reference.

### Pyocyanin extraction

*P. aeruginosa* was grown overnight in LB media and transferred to fresh LB medium at OD_600 nm_ 0.05 to initiate growth. Pyocyanin (PYO) production was measured by chloroform extraction following 24 h growth as outlined previously [43]. The extracted fraction was acidified by addition of 0.2 N HCl and measured at Abs_520 nm_ for quantification.

## Results

### Genetic variance of LTTRs within *P. aeruginosa*

~260 k LTTR protein sequences were captured from all available 699 draft, finished and permanent *P. aeruginosa* genomes from IMG using the following accession numbers: pfam00126, pfam03466 and COG0583. Redundant sequences were then removed based on knowledge of the average length of an LTTR sequence and its binding domains, by removing any sequences less than 150 amino acids and greater than 350. Truncated LTTR sequences were also removed following this approach. This resulted in ~86 000 LTTR sequences in total.

**Fig. 1.**
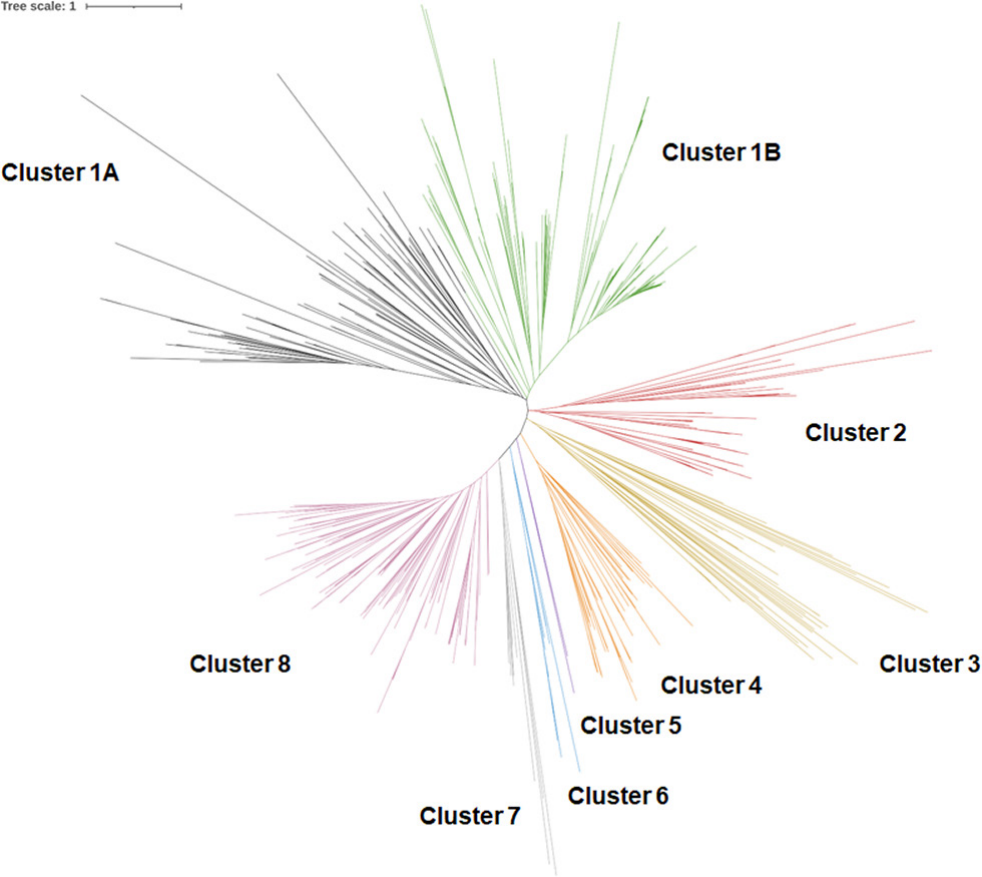
iTol visualization of a cluster tree showing the topology of the 748 LTTR representative proteins. The LTTRs can be broken up into eight distinct clusters, the largest of which was separated into two clusters, 1A and 1B. Cluster designation was selected based on nodes in the tree and colors were arbitrarily assigned for visualisation. The tree scale was large at 1, suggesting a significant evolutionary distance between each of the representative proteins.

A CD-Hit approach collapsed these sequences into 748 groups based on similarity using a 95 % threshold. As a consequence of this threshold, homologues of the same LTTR that have diverged >5 % from the consensus sequence will classify as a distinct node. Each of the 748 groups had one representative sequence, which was chosen by the programme based on a greedy algorithm, and which we subsequently used to create a cluster tree (Fig. 1 and File S1, available in the online version of this article). A muscle alignment was performed to align all of the unique LTTRs, and the tree was created using Phyml. A total of eight distinct clusters were assigned to the tree and the largest cluster, cluster 1, was split into two clusters 1A and 1B.

Analysis of the resulting tree was then performed to understand the grouping of the LTTRs into this specific topology (Table 1). Cluster 1 combined represented the greatest number of LTTR proteins, covering 42.2 % of the total number of LTTR homologous identified in the 699 genomes. Cluster 8 was the second largest of the clusters, representing a further 27.18 % of the *P. aeruginosa* LTTR protein cohort. On the other side of the scale, clusters 5, 6 and 7 were by far the smallest, representing 0.05, 1.59 and 2.42 % of the total LTTR repertoire in the genomes investigated.

**Table 1.** Table showing the distribution of the individual LTTRs into eight distinct clusters and the number of homologues they represent in total. The table represents the number of unique LTTRs per cluster and the total number of homologous members represented by the cluster overall

Cluster no.	1A	1B	2	3	4	5	6	7	8	Total
No. of distinct LTTR-encoding genes	123	214	84	44	68	2	9	14	190	748
N (Homologues)	19 194	16 726	10 586	4198	7996	41	1361	2073	23 314	85 789
% (Homologues)	**22.72**	**19.5**	**12.34**	**4.89**	**9.32**	**0.05**	**1.59**	**2.42**	**27.18**	**100**

### Intra-cluster analysis of the LTTR cohort

The vast majority of LTTRs are yet to be characterized in the *P. aeruginosa* genome, with the function and targets of only 22 out of 748 (3 %) currently known with confidence based on gene annotation. This highlights the need to elucidate conserved patterns of distribution and sequence conservation, such that experimental design underpinning efforts to characterize these proteins can be better informed.

#### Cluster 1A

A large cluster containing 123 unique LTTRs, which represents over 22 % of the total number of homologous LTTRs in *P. aeruginosa*, several members of this cluster have previously been characterized by virtue of their role in pathogenesis and biofilm formation. Characterized members include MexT, which is linked to the MexEF-OprN efflux system and is also known to regulate genes involved with antibiotic resistance [9, 44]; DguR, which is a regulator of the *dguA* promoter and involved in D – Glu catabolism [45]; YeiE, which regulates *lysP* expression [46]; PcaQ, which regulates genes involved with the catabolism of phenolic compounds [47]; and PA2206, which regulates genes involved with pathogenesis and oxidative stress [48]. In fact, the binding motif identified for the PA2206 LTTR was also found to exhibit similarity with the PcaQ binding motif, perhaps a reflection of their similar evolutionary trajectory [48]. PA2121 (biofilm synthesis repressor BsrA) has recently been shown to be involved in early biofilm formation, mature biofilm development, and colony morphology in *P. aeruginosa* [49, 50]. Specifically, the protein product of *PA2121* was found to repress the biosynthesis of extracellular polysaccharides (alginate, Psl and Pel). PA5437 (PycR) has been shown to be essential for maintenance of *P. aeruginosa* in the rat lung, controlling expression of the *pycA* pyruvate carboxylase locus [51].

#### Cluster 1B

This cluster contains the largest amount of unique LTTRs in this study, with 214 in total being represented here. However, it only represents 19 % of the total number of LTTR homologues in the pathogen. Therefore, while it captures much of the diversity of LTTR proteins in *P. aeruginosa*, these individual proteins (i) may have been less successful in achieving widespread distribution within the species or (ii) may have undergone divergence leading to small numbers of variants with less than 95 % identity to the representative sequence. A variety of categorized LTTRs can be found in this cluster, with various functions including MetR, which is involved in swarming [52] and controls genes involved with methionine synthesis [53]; BauR, which regulates genes involved in the catabolism of polyamines [54]; OxyR, the global regulator of oxidative stress [11]; CysB, the global regulator of sulphur metabolism [55]; CynR, which regulates the *cyn* operon needed for the production of the enzyme cynase, which catalyzes the decomposition of cyanate [56]; CatR, which regulates the *catBC* operon allowing the pathogen to grow on benzoate [57]; AlsR, the regulator of the *alsSD* operon, which is essential for a response to acetate accumulation in anaerobic conditions [58]; DhcR, which is required for the catabolism of carnitine, an energy source for the pathogen when establishing an infection [59]; GbuR, which regulates the *gbuA* gene, which is involved in the arginine dehydrogenase pathway [60]. An interesting corollary between clusters 1A and 1B was the presence of a large abundance of LTTRs involved in regulating the response of *P. aeruginosa* to oxidative stress.

#### Cluster 2

Cluster 2 contains 84 unique LTTRs and represents 10 586 homologous LTTRs in total. PqsR is found in this cluster, which is involved with various functions including QS and pathogenesis [8] and NmoR, which regulates the transcription of *nmoA,* which is involved in the detoxification of 3-nitropropionic acid [61]. PqsR controls part of the QS regulon in *P. aeruginosa*. While the two co-inducer signal molecules HHQ and PQS have been shown to possess interspecies and interkingdom activity, and HHQ has been shown to be produced by other species of bacteria such as *Burkholderia cenocepacia* and some *Aeromonas* species, *pqsR* homologues have not been identified outside of the *P. aeruginosa* species. PA3225 is a repressor of the *PA3225-PA3228* operon that has been associated with biofilm-mediated antibiotic resistance and type VI secretion [62]. PA1413 has recently been shown to regulate biofilm and *in vivo* pathogenesis in concert with another LTTR protein PA1226 [63]. More recently, a small RNA encoded downstream of *PA1413*, namely *sicX* (*PA1414*)*,* highly expressed in human chronic wound and cystic fibrosis infections, has been shown to play a key role in the switch between chronic and acute infection [64].

#### Cluster 3

This cluster contains 44 representative LTTRs and represents nearly 5 % of the total homologues in the pathogen. MdcR is present in this cluster, which is a possible regulator of the *mdcABCDEFGH* cluster involved with malonate decarboxylase [65]. PA2076 (OdsR) has recently been characterized as an oxylipin receptor protein governing a form of fatty acid mediated quorum sensing in *P. aeruginosa* [66]. The other LTTRs in this cluster remain uncharacterized.

#### Cluster 4

This cluster has 68 unique LTTRs and represents 7996 LTTRs in total. AmpR the well-characterized LTTR, which plays a major role in antibiotic resistance through regulation of the *ampC* gene [67], is found in this cluster. Also found here is TrpI, which regulates the *tryBA* gene pair required to produce the enzyme tryptophan synthase [68]. GdcR, which is involved with glutarate utilization and is part of a lysine catabolic network in *P. aeruginosa*, is also present in this cluster.

#### Cluster 5

This is the smallest cluster containing only two distinct LTTR sequences, neither of which are characterized, representing only 41 out of total of 85 789 sequences.

#### Cluster 6

This is the second smallest cluster containing nine unique LTTRs. However, it still represents 1361 homologous sequences out of a total 85 789. ArgP, also known as IciA, which is involved in the repression of DNA replication and amino acid metabolism [69, 70] is included in this cluster.

#### Cluster 7

This cluster has 14 unique LTTRs representing 2073 homologous LTTRs in total. All LTTRs present in this cluster remain to be characterized.

#### Cluster 8

This cluster has 190 LTTRs representing 23 314 homologous LTTRs, which is over 25 % of the total homologues, making it the largest single cluster after the combined cluster 1. However, despite this, the only characterized members are BexR, which is a bistable expression regulator [71], GfnR, which regulates the glutathione-dependent formaldehyde detoxification system in *P. aeruginosa* during the catabolism of sarcosine [72], PtxR, which regulates the genes involved with exotoxin A production [73], and BvlR, which is required for full virulence and tight microcolony formation in *Pseudomonas aeruginosa* [10]. Therefore, there are still 187 unknown LTTRs in this cluster alone based on a 95 % identity threshold, further evidence of the dearth of understanding around how this complex network of transcriptional regulators operates at strain and population level.

### LTTR distribution across and within the *P. aeruginosa* genomes

The number of homologues found in the assigned clusters ranges from a low of 41 in cluster 5 to a high of 23 314 in cluster 8. Therefore, we next explored how the individual LTTRs are distributed across the 699 sequenced strains of the pathogen. When identifying the various characterized LTTRs on the cluster tree, it was evident that some LTTRs have diverged by more than that allowed by the 95 % threshold. Therefore, to accurately measure the distribution of each LTTR protein as an entity across the genomes, and to account for this diversity, a CD-Hit using an 80 % threshold was carried out to ensure that even diversifying homologues were captured under a single representative LTTR. We reasoned that all functional LTTR protein homologues would exhibit >80 % sequence identity, even allowing for those with the greatest diversification. This resulted in a total of 415 unique LTTRs representing the ~86 000 homologues (File S2).

**Fig. 2.**
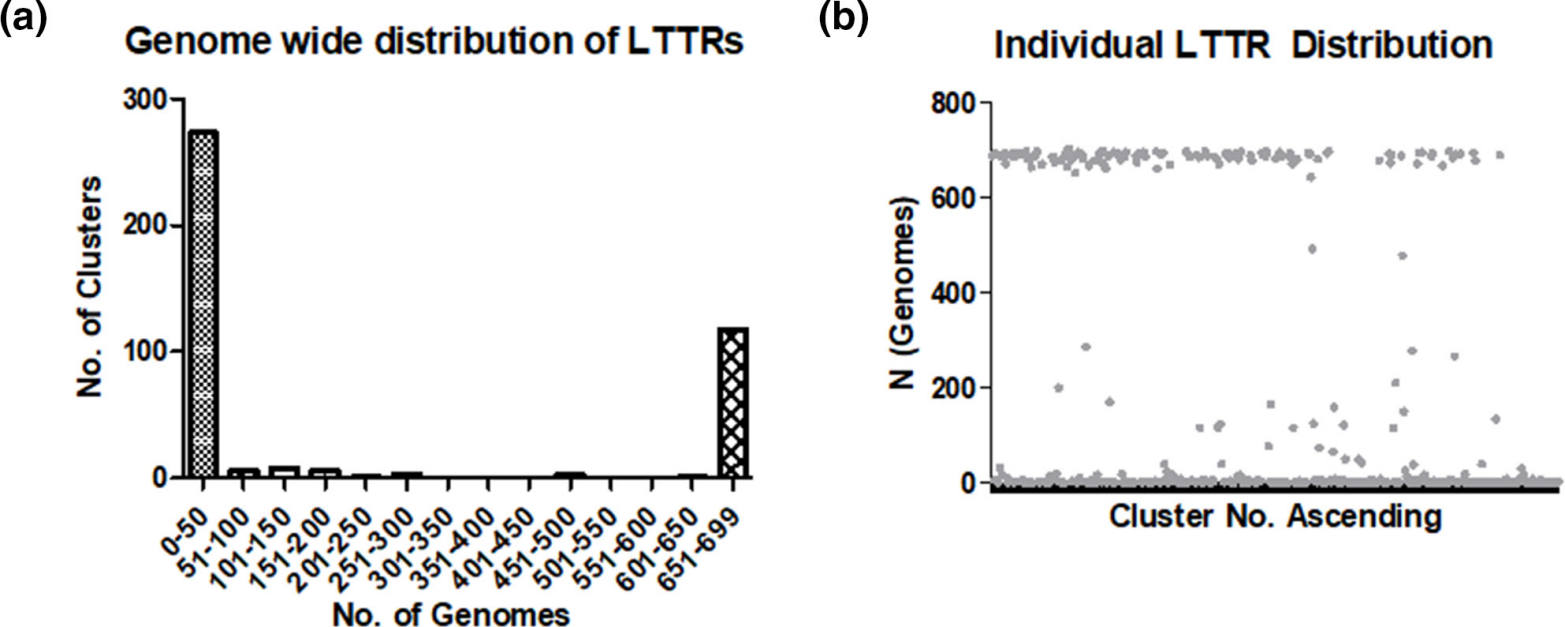
(**a**) Histogram representing the distribution of *P. aeruginosa* genomes per cluster number. The distribution of LTTR proteins across the *P. aeruginosa* genomes suggests an either/or occurrence whereby a large number of LTTRs are present in less than or equal to 50 genomes, with a very small amount present in more than 50 and less than 650 genomes. Approximately 25 % are present in over 650 genomes. (**b**) Scatter plot showing the number of genomes each LTTR is present in. Consistent with the distribution plot, a large number of LTTRs are only present in less than ten genomes. Images created using ggplot2.

The distribution of these 415 LTTRs was then analyzed, revealing a very interesting frequency across the sequenced strains. The majority of the LTTRs encoded in the *P. aeruginosa* species are either distributed in ≤50 genomes, or >650 genomes (Fig. 2a). There is very little evidence of distribution at the level of 50–650 strains suggesting an ‘either’ ‘or’ profile to the frequency of distribution. We considered those LTTRs distributed in >650 genomes (93 % of the assembled dataset) to be core, identifying 117 ‘core’ LTTRs that fall into this category (Fig. 2b). Of these, the majority were encoded in clusters 8(28 %), 1A (23%), 1B (18%) and 2(13 %), with less prevalence observed in the remaining clusters [4(9.6 %), 3(5.3 %), 7(2.6 %), and 6(1.7 %)]. No core LTTRs were found in cluster 5. We also considered the frequency with which core LTTRs are represented in each cluster, and here we observed a very different pattern. When normalized based on the size of the individual clusters [(% core LTTRs/number of cluster members)*100], there was a far more even spread of core LTTR representation in the clusters [6(19.5 %), 7(19.0 %), 1A (19.0 %), 2(16.0 %), 8(14.3 %), 4(14.1 %), 3(12.0 %), 1B (8.2 %)] with the exception of 1B, which contained a much lower representation of core LTTRs within its members.

More than half of what were considered to be ‘accessory’ LTTRs (i.e. distributed in less than 7 % of available genomes, *n*=297) have less than ten homologues within *P. aeruginosa*. This U-shaped distribution whereby the LTTR encoding genes are either common or rare is consistent with previous studies on the accessory genome [74]. It is worth noting that while many of these LTTRs are infrequent within *P. aeruginosa*, in some cases they appear to be distributed in other non-*Pseudomonas* species of bacteria, and thus the distribution here may simply reflect acquisition or transfer of LTTRs among species of co-colonizing bacteria. It follows, therefore, that a more extensive analysis of the evolutionary history of LTTRs is required before these patterns could be considered species-wide.

**Fig. 3.**
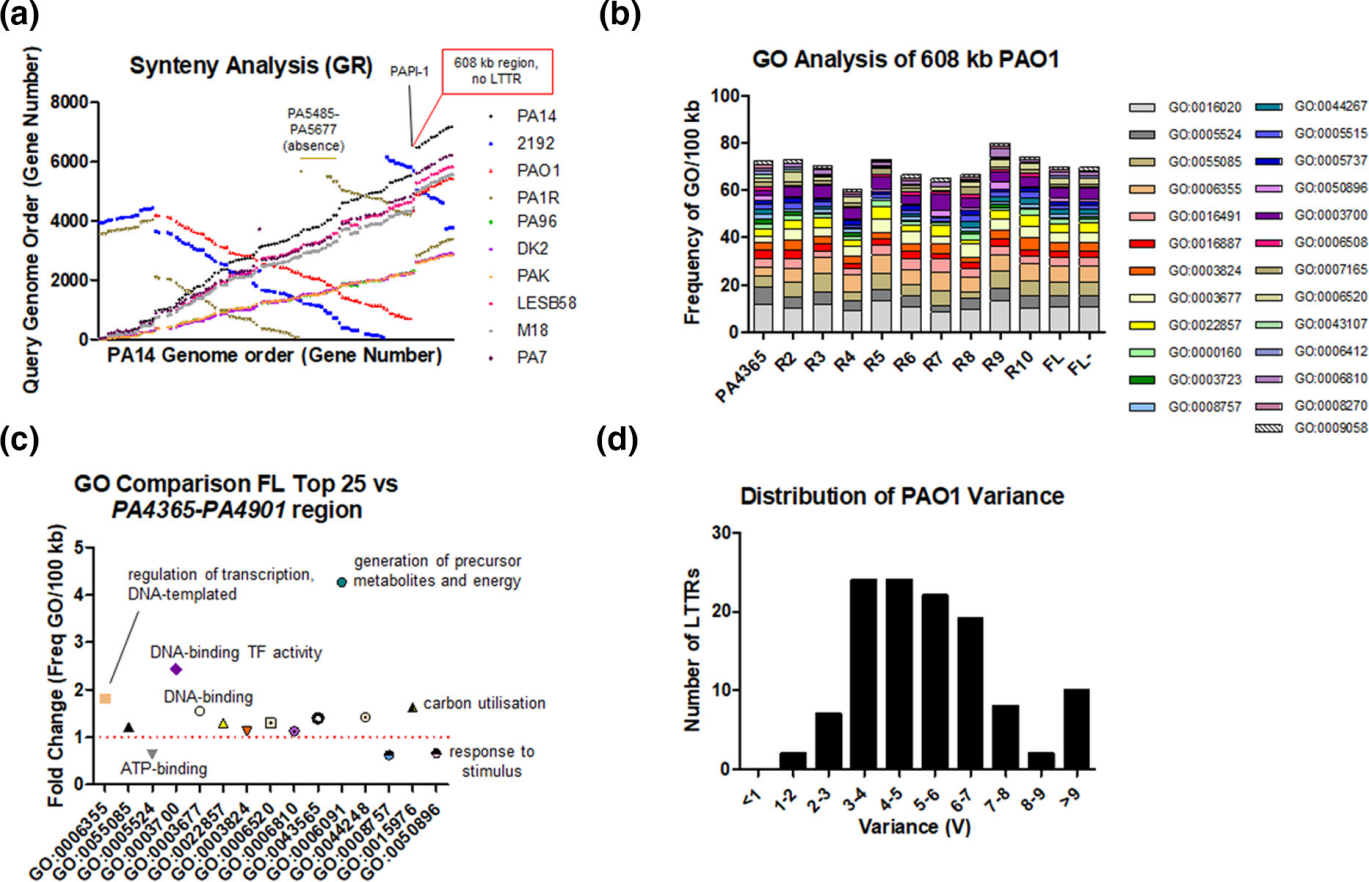
Synteny, ontology and variance frequency distribution taking a (**a**) PAO1 rooted approach. (**a**) Synteny analysis of the PAO1 LTTR repertoire compared to the 2192, PA14, and PA1R genomes, with gene order on the *x*-axis provided by the PAO1 genome. The *y*-axis represents the gene designation number (in the case of PA14, PA96, DK2, PAK and LESB58, this has been divided by 10 to align with the other represented genomes). Several regions of interest are noted in the figure, including a 608 kb region in which no LTTR encoding genes were identified (spanning the PAPI-1 site in PA14). (**b**) The top 25 GO assignments for the 608 kb region from *PA4365-PA4901* are shown in comparison with other 608 kb regions (R2, *PA4902-PA5428*; R3, *PA5429-PA0411*; R4, *PA0412-PA0977*; R5, *PA0978-PA1529*; R6, *PA1530-PA2072*; R7, *PA2073-PA2555*; R8, *PA2556-PA3112*; R9, *PA3113-PA3664*; R10, *PA3665-PA4207*) and both the full genome (FL) and full genome with the 608 kb LysR-free region (*PA4365-PA4901*) removed (FL-). The *y*-axis represents the frequency of each GO term per 100 kb in a stacked chart formation. (**c**) Fold change in the top 25 GO terms in the PAO1 genome (FL) relative to the *PA4365-PA4901* region. Apart from the GO terms described above in (b), GO0006091 (generation of precursor metabolites and energy) and GO0015976 (carbon utilization) presented the highest increased frequency per 100 kb in the FL relative to the LysR-free region. (**d**) Histogram showing the frequency of variance across the entire PAO1 repertoire with the *x*-axis representing the degree of variance and the *y*-axis representing the number of LTTRs per designation.

When the relative order of LTTRs was investigated in the genome comparisons it was evident that it followed a syntenic distribution (Fig. 3a). Rearrangements in the order of LTTRs reflected wider genome re-organization in the *P. aeruginosa* genomes [75, 76]. Intriguingly, a large region comprising 608 180 bp (*PA4365-PA4901*) was observed in which no LTTR-encoding genes were identified. Gene ontology (GO) analysis of this region identified a shift in function when compared with the full genome (FL) or with other similar sized regions of the genome (Fig. 3b). GO terms for which there was a marked increased frequency in the *PA4365-PA4901* region compared to subsequent 608 kb regions 2–10 (R2-R10) and FL/FL- include GO0005524 (ATP binding), GO0003723 (RNA binding), GO0008757 (S-adenosylmethionine-dependent methyltransferase activity), GO0050896 (response to stimulus), GO0006508 (proteolysis), GO0007165 (signal transduction), GO0043107 (type IV pilus-dependent motility), GO0006412 (translation) and GO0008270 (zinc ion binding). In contrast, in the same comparison there was a marked reduction in GO0006355 (regulation of transcription, DNA-templated), GO0003677 (DNA binding), GO0003700 (DNA-binding transcription factor activity), GO0022857 (transmembrane transporter activity) and GO0006520 (cellular amino acid metabolic process). When compared to the top 25 most abundant GO terms in the full genome, a similar profile was observed, with GO0006091 (generation of precursor metabolites and energy), and GO0015976 (carbon utilization) also exhibiting a marked reduction in frequency per 100 kb in the LysR-free region (Fig. 3c). The reduction in frequency of several GO terms relating to transcriptional regulation would suggest that the observation is not an LTTR-specific one, but rather may relate to a wider phenomenon within this 608 kb region of the *P. aeruginosa* genome.

### Genetic variance of core LTTRs across the *P. aeruginosa* genomes

In order to better understand the evolutionary trajectory of the core LTTR sequences, CD-Hit using a 100 % threshold was carried out on each of the 118 core LTTR groups. This enabled us to study how conserved or varied the LTTR is across all of its homologues. Homologues with identical sequences grouped together into clusters, therefore allowing us to analyze the evolution of these proteins, with PAO1 used as the baseline sequence for representation. The PAO1 strain, originating from a wound isolate in Melbourne Australia, is one of the model strains used widely in functional and phenotypic *P. aeruginosa* studies, being the first to have its genome sequenced to completion [77]. It remains one of the best annotated genomes available for *P. aeruginosa* and it provides a useful context with which to interpret the literature where PAO1 annotations largely predominate.

The variance of each of the individual LTTRs was calculated by dividing the number of ‘clusters’ of identical sequences (Vn) by the total number of homologues in the sequenced genomes (Sn). The mean variatiotn was calculated across the core LTTRs as 5.4 % and the median was calculated as 5.1 %. Variance frequency followed a U-shaped distribution, whereby the lowest and highest values, representing the most and least conserved LTTR proteins, were 1.43 and 12.10 %, respectively (Fig. 3d). The core top ten most conserved and top ten most variable LTTRs (Table 2) were then identified and subjected to further analysis in order to gain insights into the nature of the conserved vs variable structure of LTTRs within the species. It was interesting to note that several of the most conserved LTTR proteins have previously been shown to play a role in protecting the cell from oxidative stress, a fundamental core action of the cell in host–microbe and microbe–microbe interactions. OxyR and CysB had variance values of 1.43 and 2.15 %, respectively, compared to PA0479 and PA0708, which had values of 12.1 and 11.8 %, respectively. The suggestion that significant constraints are placed on the highly conserved LTTR proteins would be consistent with the concept of gene essentiality and by extension, the relative flexibility of the least conserved may reflect a form of niche-specialisation or indeed co-evolution [78].

**Table 2.** Ten most conserved and most variable core LTTRs

Gene ID	Cluster	Sn	Vn	V	Gene ID	Cluster	Sn	Vn	V
	Least variable, most conserved				Most variable, least conserved				
OxyR	1B	698	10	1.43	PA0479	2	686	83	12.1
PA0528	1B	699	13	1.86	PA0708	2	678	80	11.8
CysB	1B	699	15	2.15	NmoR	2	668	75	11.2
PA5437	1A	696	15	2.16	PA2547	8	687	77	11.2
PA3122	1A	695	17	2.45	PA3776	1B	687	66	9.6
PA3398	1A	693	17	2.45	AmpR	4	694	66	9.5
BauR	1B	696	19	2.73	PA0477	1A	694	66	9.5
PA0876	8	695	20	2.88	PA0816	1A	688	64	9.3
MetR	1B	694	20	2.88	PA1826	2	665	61	9.2
PA2551	1A	697	21	3.01	PA1399	1A	683	62	9.1

Sn, Number of strains encoding LTTR; V, Variance value % defined as Sn/Vn * 100; Vn, Number of variants identified through CD-hit analysis.

#### Ten most conserved core LTTRs

The ten most conserved core LTTRs included OxyR, CysB, MetR, BauR and six uncharacterized LTTRs. These are present in nearly all of the available genomes and they have remained highly conserved across all, in spite of the widespread distribution (Files S3 and S4). Additionally, it was interesting to note that all of the most conserved LTTR proteins were found in clusters 1A and 1B of the original representative tree (Fig. 1 and File S1) with one exception. The uncharacterized LTTR with locus tag PA0876 can be found in cluster 8, the second largest cluster.

OxyR is the least variable LTTR in *P. aeruginosa*, which is not surprising as it is a global regulator of oxidative stress, while CysB is the master regulator of sulphur metabolism. PA0528 is located next to the small regulatory RNA *rsmY*, which is involved in post-transcriptional regulation [79].

#### Ten most variable core LTTRs

In contrast, the ten most variable LTTRs exhibited markedly different distribution and variance, with little evidence of a single dominant variant (Files S3 and S4). The only characterized LTTR in this group is AmpR, a global regulator of pathogenesis in *P. aeruginosa* [7]. While the remaining LTTRs in this group are yet to be characterized, it is interesting that two of the most variable LTTRs, PA0477 and PA0479, are co-located in the genome. Additionally, in contrast to the conserved LTTRs, the most variable LTTRs are spread across a variety of clusters including 1A, 1B, 2, 4 and 8, with cluster 2 predominating.

### Selective pressure across the most variable core LTTRs

Previously, we have shown that the topology of LTTR encoding genes is conventional with respect to the divergent orientation of LTTR encoding protein and the adjacent gene [5]. To explore further the type of arrangement and determine whether or not some pattern would exist with respect to most conserved and least conserved genes, we undertook a topographical analysis of LTTR distribution in the PAO1 strain (File S5). As expected, 103 of the LTTR encoding proteins were oriented divergent to the adjacent gene, though it should be noted that this does not necessarily represent the target gene/promoter of the LTTR protein. A further ten LTTR encoding genes were oriented in series with adjacent genes, while the remaining four genes were oriented facing either the preceding or the next gene in series. An interesting feature of the LTTRs exhibiting the ‘in series’ non-classical topology (i.e. no shared promoter), was that they were found in close associations in the cluster tree, e.g. PA0159, PA2123, PA2681, PA2838 and PA4145 (cluster 8), as well as PA0233 and PA3878 (cluster 1B) (Fig. 1 and File S1.

In a previous study, five of the top ten conserved LTTRs (*oxyR*, *cysB*, *metR*, *PA2551* and *PA3398*) had shown significant evidence of positive selection [5]. Therefore, the McDonald–Kreitman test was carried out on the most variable core LTTRs to identify if there was evidence of selective pressure underpinning the observed variability. Variation within sequences was compared to that with an appropriate outlier, which was selected from the same genus as the subject sequence [36]. Evidence for positive selective pressure (*P*<0.05) was detected in *PA0479*, *PA1826* and *ampR*, using a 95 % confidence interval (Table 3).

**Table 3.** McDonald–Kreitman test analysis of the ten most variable core LTTRs

Gene ID	V	Outlier	Ps	Pn	Ds	Dn	Neutrality Index (NI)	*P*-value
**PA0479**	12.1	*P. fluorescens* strain NCTC10783	19	18	24.4	76.2	0.303	0.002
**PA1399**	9.1	*Pseudomonas sp*. AK6U	5	8	10.9	18.5	0.938	0.925
**PA1826**	9.2	*Pseudomonas sp*. AK6U	32	88	87.1	120.6	1.987	0.005
**AmpR**	9.5	*Pseudomonas sp*. AK6U	17	11	17.7	46.0	0.249	0.002
**PA0477**	9.5	*P. fluorescens* strain NCTC10783	10	11	20.2	35.9	0.616	0.349
**PA3776**	9.6	*P. fluorescens* strain NCTC10783	4	7	21.9	20.8	1.842	0.377
**PA2547**	11.2	*P. fluorescens* strain NCTC10783	5	7	14.3	49.1	0.407	0.163
**PA0816**	9.3	*P. fluorescens* strain NCTC10783	12	15	0	0	NULL	NULL
**PA4203**	11.2	*P. fluorescens* strain NCTC10783	1	1	1.48	0	NULL	0.307
**PA0708**	11.8	*Pseudomonas sp*. AK6U	1	6	3.55	27.3	0.779	0.837
		**Housekeeping Gene**						
**RpoD**		*P. fluorescens* strain NCTC10783	33	49	222.7	454.7	0.728	0.185

**Fig. 4.**
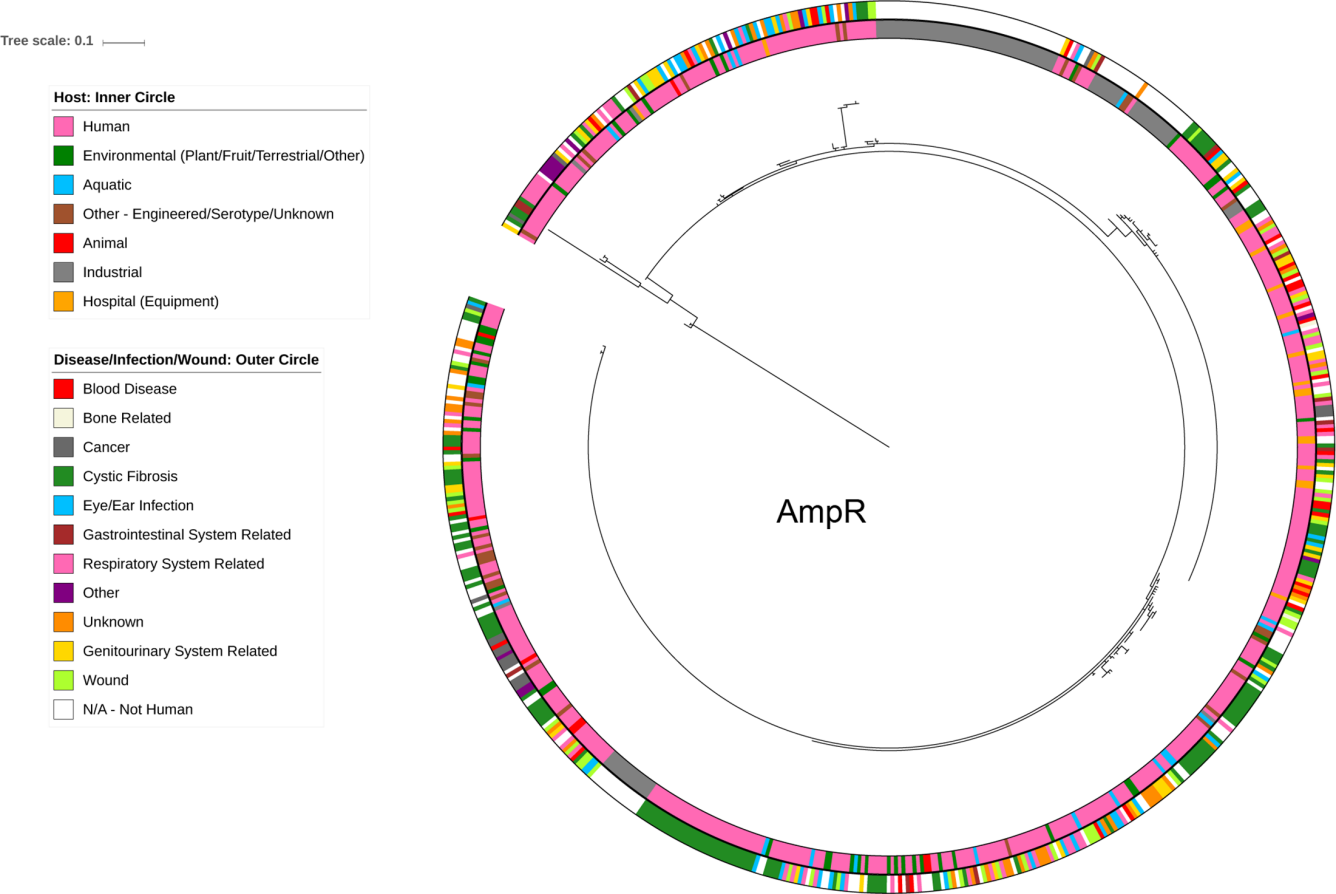
Metadata analysis of AmpR cluster tree representing most variable LTTRs. Random distribution of source across the AmpR cluster tree suggests that the environment may not be the primary selective pressure driving evolution of this LTTR in *P. aeruginosa*. The inside circle represents the host designation for each genome included in the analysis. The outer circle represents the ecosystem source context for each genome falling into categories of disease/infection/wound. Metadata trees for remaining nine most variable LTTRs are available in File S6.

*PA0816* gave a NULL result, most likely arising from the fact that the outlier sequence was too similar the *P. aeruginosa* homologue. The finding that *PA0708* did not show evidence of positive selective pressure was surprising given its high variance value. However, when metadata from the IMG database was overlaid on the cluster trees, no evidence for the basis of this selective pressure was seen. Clustering appeared to occur within the trees, independent of the source of the isolate, with clinical and environmental sources identified for homologues of the same variant (Fig. 4 and File S6).

### Distribution of the most conserved and most variable core LTTRs outside the *Pseudomonas* genus

It became evident when sourcing outlier proteins for the McDonald–Kreitman analysis, that several of the LTTR proteins under investigation were distributed outside of the *P. aeruginosa* species. Therefore, blastn nucleotide searchers were performed using (i) *‘Pseudomonas* genus, (ii) excl. *Pseudomonas aeruginosa*’ and (iii) ‘excl. *Pseudomonas* genus’ search parameters. A marked difference was observed in the frequency of LTTR distribution within the genus and outside of *Pseudomonas*. While the most conserved LTTRs were found to be distributed in other *Pseudomonas* species, and in non-Pseudomonads, the least conserved core LTTRs were rarely found outside of *P. aeruginosa*. For example, CysB and OxyR had the highest number of blastn nucleotide hits, and were found to be encoded in a broad range of other *Pseudomonas* species (Table 4). In contrast to this, several of the top ten most variable LTTRs (*PA0479*, *PA0708*, *PA2547, PA0816* and *PA1826*), while present in nearly all available strains of *P. aeruginosa* (>95 %), were only present in the genomes of two or three other *Pseudomonas* species (*Pseudomonas* sp., *P. fluorescens* and *P. paraeruginosa*). Furthermore, all of the top ten most variable LTTRs were found to be *Pseudomonas* genus specific (≥90 % query coverage, ≥70 % sequence identity).

**Table 4.** blastn analysis of the core LTTRs

	No. of blastn hits (≥90 % query coverage)				
**Ten least variable, most conserved**			**Ten most variable, least conserved**		
**Gene ID**	***P* excl. *Pa***	**Excl. *P***	**Gene ID**	***P* excl. *Pa***	**Excl. *P***
OxyR	803	73	PA0479	6	0
PA0528	63	4	PA0708	5	0
CysB	870	101	NmoR	20	0
PA5437	338	57	PA2547	5	0
PA3122	795	41	PA3776	19	0
PA3398	586	44	AmpR	196	0
BauR	62	14	PA0477	11	0
PA0876	86	0	PA0816	7	0
MetR	941	70	PA1826	8	0
PA2551	932	66	PA1399	22	1

P excl *Pa – Pseudomonas* genus excl *P. aeruginosa*; Excl P – Excluding *Pseudomonas* genus.

### An expanded PA14-centric exploration of LTTR diversification

Analysis of the available *P. aeruginosa* genome sequences from the IMG database analysis strongly suggested a form of selection in the diversification of LTTR proteins in the *P. aeruginosa* species. While informative and well curated, the Pseudomonas database offers a more expansive dataset with which to investigate the observations described above. We therefore undertook a PA14-rooted analysis of LTTR diversification. PA14, which originated from a burn wound isolate in Pennsylvania, USA, has emerged as a model of choice for infection and pathogenicity research [80], being also the source of the Harvard TnM non-redundant mutant library frequently used by the research community for functional genomics studies [81].

The topology and organisation of the LTTR-encoding genes in PA14 was first studied to determine if any unusual patterns existed relative to the conventional divergent organisation. As seen for PAO1, the LTTR-encoding genes in PA14 were predominantly arranged divergent with respect to adjacent genes, with 13 arranged in series and five arranged in opposite orientation with adjacent genes (Fig. S7). These non-classically arranged LTTRs were predominantly found on the leading strand of the *P. aeruginosa* PA14 genome. Consistent with the PAO1 genome, the distribution of LTTR encoding genes in the PA14 genome followed a syntenic pattern (Fig. 3a). In addition, the 608 kb region devoid of LTTR-encoding genes was also present in the PA14 genome, extended further as a result of PAPI-1 insertion.

**Fig. 5.**
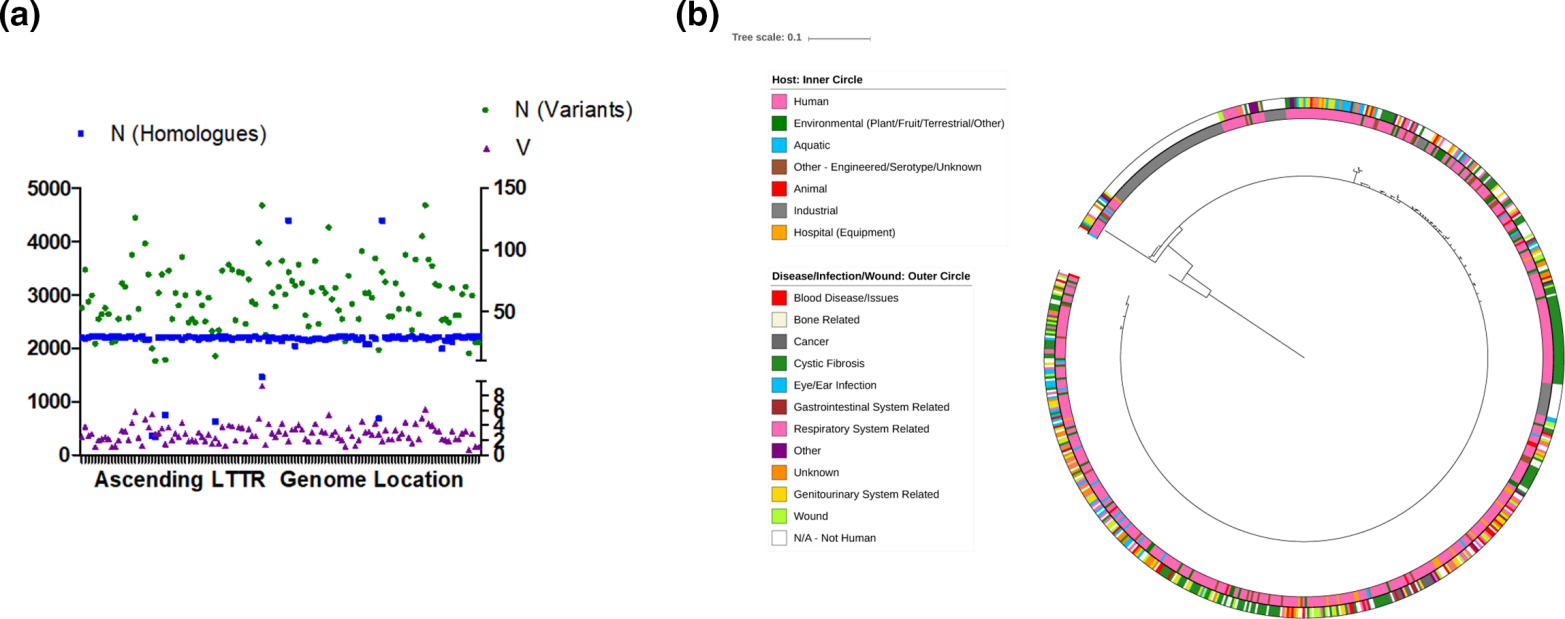
Diversification of LTTRs in PA14. (**a**) PA14-centric analysis LTTR profiles in available genome sequences. (**a**) Random distribution of variants across the PA14 genome, with LTTRs presented in ascending order (from left to right) according to genome location. The number of homologues (blue) is aligned to the left *Y*-axis, while the number of variants (green) and the V value (purple) are both aligned with the right *Y*-axis. (**b**) Phylogenetic tree of PqsR with added metadata. The inner circle represents the host and the outer circle represents infections/wounds and diseases. Due to high level of sequence conservation among the PqsR sequences, a tree with branch lengths removed for the purpose of visualising how the groups have clustered is provided in File S10.

Each of the 123 LTTRs encoded in the PA14 genome was subjected to a Diamond blastp analysis and the number of variants collated using CD-Hit. This analysis identified a total of 7748 variants comprising 265 086 homologues. Genomes for which sequencing was incomplete at the LTTR locus, and therefore contained ‘X’, were removed from the analysis to avoid the generation of spurious variants. This refinement of the database resulted in three genes (PA14_30450, PA14_40910 and PA14_32700) being removed due to a high percentage loss of original variants. The total number of variants identified in the PA14-centric cohort of 120 proteins was 7356 from a dataset of 258 194 homologues. The median number of variants was 62 and the median variance (V) was 2.81, and as with PAO1, it followed a U-shaped distribution (Fig. 5a, Table 5 and File S8).

**Table 5.** Ten most conserved and most variable PA14 LTTR proteins

Gene ID	Locus (PAO1)	Sn	Vn	V	Gene ID	Locus (PAO1)	Sn	Vn	V
Least variable, most conserved					Most variable, least conserved				
PA14_70560	OxyR	2224	16	0.72	PA14_30970		1457	136	9.33
PA14_01640	BauR	2214	24	1.08	PA14_54710	PA0191	2206	136	6.17
PA14_71640	PA5428	2223	25	1.13	PA14_06260	PA0479	2174	126	5.80
PA14_02650	PA0217	2223	25	1.13	PA14_10090		363	20	5.51
PA14_71750	PA5437	2221	25	1.13	PA14_37940	CynR	2174	118	5.43
PA14_41870	CysB	2221	26	1.17	PA14_54130	PA0784	2214	111	5.01
PA14_02660	PA0218	2217	26	1.17	PA14_29440	PA2681	2175	106	4.87
PA14_46060	GbuR	2208	27	1.22	PA14_09570	NmoR	2160	105	4.86
PA14_23730	PA3122	2223	28	1.26	PA14_46330	PA1399	2194	99	4.51
PA14_06880	PA0528	2222	28	1.26	PA14_51340	PqsR	2174	96	4.42

Several LTTRs that are only present in a subset of *P. aeruginosa* strains, exhibited a high variance (V) score [Sn/Vn)*100], signifying a high degree of variation even within such a limited number of genomes. These included PA14_30970, PA14_54710, and PA14_06260 with V scores of 9.33(136/1457), 6.16(136/2206) and 5.8(126/2174), respectively (Table 5). PA14_10060 was also noticeable in that it had the fourth highest V value of 5.51(20/363) in spite of its relatively low frequency of distribution. On the other end of the spectrum, among the most highly conserved LTTRs, V scores as low as 0.72(16/2224) were observed in PA14_70560, encoding OxyR (Table 5). PA14_10830 was interesting in that it had a relatively low V score of 1.47(11/747) despite the fact that homologues were only found in a limited number of the available genome sequences.

The congruence between the order of LTTR proteins with respect to conservation was notable when compared with the IMG database analysis described earlier. While some of the top ten highly conserved and highly variable LTTRs varied slightly between both analyzes, those that were absent from the top ten in either dataset were found within the top quartile (File S8). The striking contrast between the highly conserved and most variable LTTRs suggests that there are evolutionary pressures on this important class of transcriptional regulator family. Perhaps some of this arises from the fact that LTTRs are modular signal responsive transcriptional regulators, fine-tuned to control expression in response to substrate availability, or in some cases signal molecule thresholds. The inclusion of PqsR in the top ten most variable LTTRs was particularly interesting in light of the fact that this protein is a key control point in population level decision making in *P. aeruginosa*.

With some exceptions, homologues of each of the PA14 LTTRs were encoded in more than 95 % of the genomes investigated. The number of variants ranged from 11 to 136, and there was no evidence of a location bias to this diversity, with the possible exception of PA14_10090, PA14_10120, and PA14_10830. Following expansion of the genome dataset in the Pseudomonas database, the ten most conserved and most variable LTTR proteins were re-analyzed using the ~8000 genomes available (~600 complete). The outcome was again consistent with analysis presented above, both groups (most conserved vs most variable) exhibited significantly different variant number (Vn) and variance (V) values presented here as lower 95 % CI of mean: (69.57–83.03 vs 166.9–267.5, *P*<0.001, Student’s *t*-test) and (0.87–1.04 vs 2.61–3.85, *P*<0.001, Student’s *t*-test), respectively (File S9). It was notable that the PqsR protein was now seventh in the most variable list of LTTR proteins.

### Functional consequence of evolution of PqsR, a global regulator of pathogenesis in *P. aeruginosa*

PqsR is one of the best characterized LTTRs in *P. aeruginosa* and is currently a major drug target [82]. Therefore, it presents an excellent case study to investigate the impact evolution of this LTTR would have on the ligand-receptor-target interaction of these LTTRs in *P. aeruginosa*. A total of 96 variants were identified among the 2174 homologues representing a variance of 4.42, which was well above the mean V of 2.81. These findings were further supported by an unbiased analysis of the genomes available through the IMG database. A total of 688 homologues of PqsR were identified in 699 genomes *P. aeruginosa*, with 49 different variants identified when the CD-Hit 100 % threshold analysis was performed. The V of PqsR was calculated as 7.12, which is higher than the mean V of 5.38 found in the core LTTRS available through IMG. The availability of metadata and its curation in the IMG database led us to focus on these datasets to further interrogate the variance in the PqsR protein (Fig. 5b and File S10).

Of the 49 different variants found, two major clustering groups were identified. PAO1, the moderately virulent model strain of *P. aeruginosa* and PA14, the highly virulent model strain, fall into each of the two main groups [51, 83, 84]. The PAO1 sub-cluster represented 459 identical homologues accounting for 66 % of the total PqsR variants within *P. aeruginosa*. The PA14 sub-cluster group had 140 identical homologues, representing 20 % of the total number of PqsR homologues. This left 14 % of the total homologues split into 47 clusters, indicating a significant level of inter-strain variation within *P. aeruginosa*. Interestingly, the putative PqsR binding motif was conserved in the *pqsA* promoter region of all genomes tested, suggesting there is no co-evolution of signal-receptor and DNA target (File S11). SNPs underpinning the variation in PqsR were dominated by Val-Ala transitions, and to a lesser extent by Glu-Asp and Pro-Leu or Pro-Gln. There was no evidence for transitions at positions comprising Ile, Met or Gln. Neither was there evidence of transition to Lys or Trp. Cluster analysis of the variants revealed a low degree of divergence relative to the core PA14 sequence, with most of the variants arising from singleton SNPs (Files S12 and S13).

**Fig. 6.**
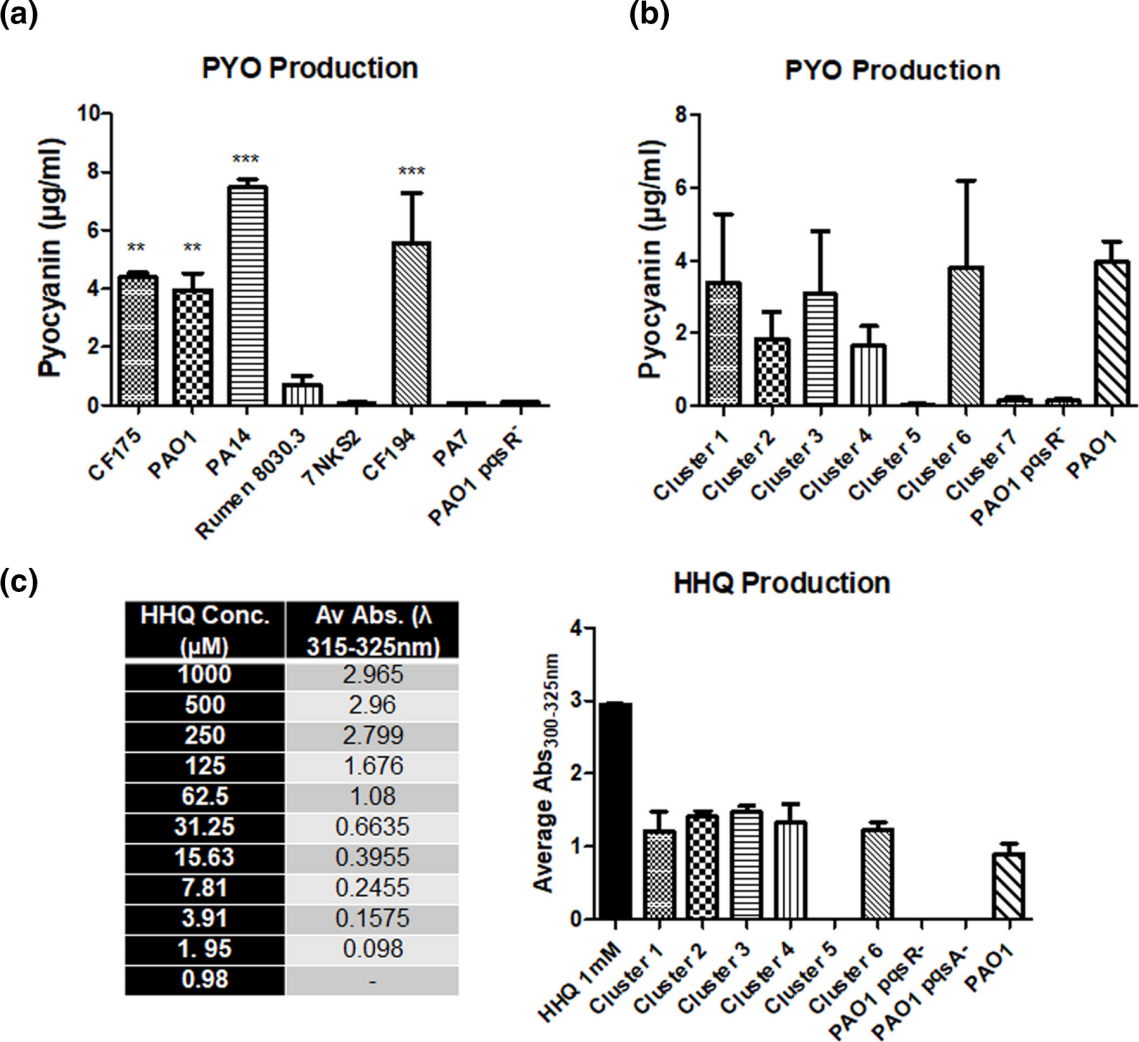
(**a**) PYO production in isolates harbouring *pqsR* variants and (**b**) PYO production in PAO1 *pqsR*- mutant complemented with each of the seven *pqsR* variant sequences. Data presented is the average of three independent biological replicates. Statistical analysis was performed by One-Way ANOVA (**P<0.005; ***P<0.001). (**c**) Spectrophotometric comparative quantification of HHQ production in standards and PAO1 *pqsR*-mutants complemented with six representative *pqsR* variant sequences. Data represented is the average of four independent biological replicates for each of the extractions.

In order to determine what impact, if any, this evolution had on the interaction between the ligand, the protein and the target promoter, the *pqsR* gene was amplified from a collection of *P. aeruginosa* isolates and sequenced. These isolates included hospital isolates and bovine rumen isolates, from which it was hoped the diversity of the system could be captured. The amplified sequences (File S14) were combined with the 688 PqsR genome derived sequences (IMG database) and concatenated into a file. CD-Hit at 100 % resulted in 52 variants of the PqsR homologues compared to the original 49 variants. Strains 7NKS2, CF194, and PA7 formed three new clusters not present in the original genome based analysis. A total of eight homologues clustered into the PAO1 group while six strains were part of the PA14 group. Interestingly, three of the rumen strains clustered together into a group which previously only had four members. The CF175, CF208 and Rumen 8043–1 sequences clustered together into another group, which previously contained only one LTTR. PqsR was then further analyzed by obtaining all of the available metadata from IMG to investigate whether or not there was any possible host and/or disease/infection associated this variance. No correlation was observed between variant and source, implying that PqsR evolution may be a mechanism for bacteria to explore new adaptations in search of finely tuned functions, rather than a response to an extrinsic source dependent need (Fig. 5b). To explore further the functional significance of the sequence variation, a representative sequence was selected from each of the seven groups of variants for which strains were available (File S14). The *pqsR* sequence was amplified from each representative strain by PCR, and the amplicons were restriction-inserted into pBBR1-MCS5 plasmid. Complementation of a PAO1 *pqsR*-mutant with these variants was assessed through pyocyanin (PYO) and HHQ/PQS production compared to wild-type PAO1 (Fig. 6 and Files S15 and S16).

Amongst the PYO producing cluster representative strains, PA14 exhibited the highest levels, with the rumen strain (representing cluster 4) being the lowest (Fig. 6a). Neither 7NSK2 (cluster 5) nor PA7 (cluster 7) produced measurable levels of PYO. Complementation of the PAO1 *pqsR*- with the variants led to restoration of PYO production at comparable levels for each cluster representative sequence, with the exception of the aforementioned 7NSK2 and PA7 sequences (Fig. 6b). As both of these latter variants appeared to be non-functional, only the 7NSK2 (cluster 5) variant was carried through as a control for the HHQ/PQS profiling analysis. Production of HHQ and PQS was analyzed by obtaining the absorbance (λ_max_) values between 200–800 nm on the UV-Vis spectrum (Fig. 6c) as well as by thin-layer chromatography (TLC, Fig. S15). HHQ’s λ_max_ was found to be at 310–325 nm. λ_max_ values between 315–325 nm were observed for each of the functional variants which correspond to the HHQ reference λ_max_ values. This method of HHQ detection was further supported by extractions from PAO1 *pqsR*- and PAO1 *pqsA*-mutants, which are incapable of HHQ biosynthesis (Fig. S16), showed no characteristic HHQ λ_max_ values. Taken together, variants from clusters 1, 2, 3, 4 and 6 were capable of restoring both PYO and HHQ production in the PAO1 *pqsR*-mutant. The 7NSK2 strain itself did not produce PYO or HHQ, and this was consistent with the presence of a frameshift mutation in *pqsR* identified in the sequence analysis. To determine whether non-functional mutations would also exist in the downstream systems involved in HHQ/PQS and PYO production in the 7NSK2 strain, we introduced a *pqsA*-lacZ reporter fusion, which revealed that there was no *pqsA* promoter activity in the strain, in the presence or absence of synthetic PQS (File S17). To explore this further, a PAO1 *pqsR* overexpression pME6032 plasmid was introduced into the 7NSK2 strain and induced by the addition of IPTG. However, PYO production was not restored suggesting that loss of PYO production is also a consequence of factors downstream of the PqsR protein in this strain. Finally, addition of synthetic PQS to the 7NKS2 strain also failed to induce PYO production, further evidence that the absence of PYO production in this strain may not be due to variation in the PqsR protein alone and that the diversification observed at the LTTR level in this study might also extend to downstream nodes in the signal transduction systems.

## Discussion

*P. aeruginosa* is currently amongst the most prominent pathogens globally from the perspective of clinical management of infection and is considered by the WHO as a top priority for research and development since it is part of the multidrug resistant group of ESKAPE pathogens [80, 85, 86]. The mechanism by which *P. aeruginosa* adapts to its surroundings relies, in part, on the use of transcription factors, with LTTR proteins being amongst the most abundant type in this pathogen [2, 5]. The manner in which such a broad spectrum of these LTTRs have arisen within *P. aeruginosa* remains unclear.

Rather than focusing solely on the individual LTTR repertoires one must consider the transcriptional unit, which despite its classical divergent organization in the case of LTTRs, may not always be apparent. The mechanisms of activation and repression used by LTTR proteins are more complex than its classical mechanism may suggest, with independent and cooperative transcriptional control reported in some cases [87]. In several well-characterized cases, the adjacent gene does not appear to be the target of the LTTR, e.g. PqsR and MexT, and therefore the topographical arrangement presented here should be viewed in that context. In some cases, the apparent existence of orphan LTTR proteins, may suggest that some operate through previously uncharacterized mechanisms, as is the case with the emerging function of LuxR solos elegantly described by Venturi and others [88, 89]. Some evidence to this effect may be seen in exploratory studies performed in *E. coli*, whereby hetero-oligomerization was observed *in vitro* [90]. Were this to be the case *in vivo*, clustering of LTTRs may reflect patterns of association between interacting protein partners, rather than a common function or signal interaction, or both. In the absence of empirical evidence, no concrete assertions can be made at this time.

*P. aeruginosa* has been well characterized through comparative genomics analysis, particularly in the case of clinical isolates where genome rearrangements and acquisitions have been noted [75, 76, 91]. While the footprint of horizontal gene transfer (HGT) is evident in strains, e.g. PAPI-1 in PA14, there has been some suggestion that HGT has not played a significant role in the evolution of the Pseudomonas genus [92]. There can however be selective pressure on the existing gene repertoires, and this can vary depending on whether the genes are part of the core or accessory genome [93]. In addition to the five genes from the top ten most conserved *P. aeruginosa* LTTRs that have previously been shown to present significant MKT outcomes [5], three genes (*PA0479*, *PA1826* and *ampR*) were identified amongst the top ten most variable LTTRs with potential for positive selection. However, it should be noted that significant bias exists within the MKT testing, not least owing to its sensitivity to the presence of weakly deleterious mutations, and the influence of recombination events where several new variants can be brought into a single lineage [94–96]. Further investigation is warranted across the *P. aeruginosa* pangenome before the true influence of selective pressure on LTTR evolution can be determined.

Previous studies have shown that the highest degree of positive selection within the transcriptional unit may reside in synvergon spacers, described as the space between consecutive genes from the same strand. Divergon spacers (common 5′ regions), such as those characteristic of the LTTR arrangement, were the next most prevalent form [97]. The importance of this, along with the evolutionary driver of leading vs lagging positioning of coding genes (itself a topic of some debate) remains to be determined [98, 99]. It was also interesting to note that the most conserved LTTRs were broadly distributed at genus level, whereas the least conserved appeared restricted to *P. aeruginosa*. This implies that the most variable LTTRs may have evolved in *P. aeruginosa* to serve a specific function in this versatile and prevalent pathogen, or that the lack of evolutionary constraint placed on these least conserved LTTR encoding genes is such that divergence has exceeded the thresholds used for comparative sequence analysis in this study. The role of niche specialization in shaping the distribution of LTTR proteins at the strain level will be particularly interesting to explore [100, 101]. In this regard, one must also consider the emerging role of intra-genomic selective pressures in determining the composition of prokaryotic genomes and that these niche-adapted genes may have positive or negative effects on other genes within the pangenome [102, 103]. The absence of LTTR encoding genes from a 608 kb region of the *P. aeruginosa* genome will be fascinating to investigate for such patterns of exclusion and inclusion.

The cluster patterns that emerged from the maximum-likelihood analysis presents some indication of functional conservation, but also serve to highlight the relatively uncharacterized nature of LTTR proteins in this species. Nine of the ten most conserved LTTRs were present in clusters 1A or 1B, while cluster 2 contained four of the ten most variable LTTRs. Cluster 2 represents 12 % of the total homologues present in the genomes used in this study, and the co-localization of such a large number of uncharacterized variable LTTRs suggests an evolutionary pattern of interest. The presence of several LTTR proteins with a role in the oxidative stress response and aromatic compound degradation in cluster 1 may be significant, and may also serve to underpin the predominance of conserved LTTRs in these clusters. Cluster 8, in spite of the fact that it represents 27 % of the total number of homologues across all genomes in this study, is predominantly uncharacterized with the exception of BexR, GfnR and PtxR. Similarly, the LTTR proteins in clusters 5 and 7 are also uncharacterized, though they represent a combined total of 2.3 % of homologues.

Complementation analysis suggests that PqsR variance is tolerated within the AQ signalling system, to a certain extent, although subtle effects not evident in the proxy assays of PYO and PQS/HHQ production performed here may underpin a more refined role. It is of course possible that the same level of sequence flexibility may not extend to other LTTR proteins exhibiting a high degree of variance. Understanding the extent to which sequence variance is tolerated, and equally why a considerable subset of LTTR proteins appear not to tolerate variance, will be important in understanding how these networks operate. It should also be noted that diversification may also occur at a sub-population level and the relevance of this within polymicrobial communities warrants further study.

Mapping the evolution of LTTRs in *P. aeruginosa* will give a better understanding of the network through which these important transcriptional regulators operate. Conformational studies that consider the degree of variance among strains of LTTRs are needed, particularly where they represent targets for small molecule therapeutics. A deeper understanding of the genomic variation at the species and strain level will facilitate the development of more refined therapies, while also improving responses to existing therapies where receptor variation is evident. Additionally, the industrial and biotechnological relevance of LTTRs underpins the importance of elucidating their function and organization.

## Supplementary Data

Supplementary material 1

Supplementary material 2
