# Supplementary material for: Comparative genomics reveals distinct diversification patterns among LysR-type transcriptional regulators in the ESKAPE pathogen Pseudomonas aeruginosa
Source: Microb Genom. 2024 Feb 29;10(2):001205. doi: 10.1099/mgen.0.001205 (PMC10926688; doi:10.1099/mgen.0.001205)
Supplement: Supplementary material 2 [file mgen-10-1205-s002.pdf]

Microbial Genomics: Supplementary Data Files

Comparative genomics reveals distinct diversification patterns among LysR-type transcriptional regulators in the ESKAPE pathogen *Pseudomonas aeruginosa*

Jamie Deery[1¥], Muireann Carmody[1,2¥], Rhiannon Flavin[1], Malwina Tomanek[1], Maria O'Keeffe[1], Gerard P. McGlacken[2,3], and F. Jerry Reen[1,3*]

[1] School of Microbiology, University College Cork, Cork, Ireland.

[2] School of Chemistry, University College Cork, Cork, Ireland.

[3] Synthesis and Solid State Pharmaceutical Centre, University College Cork, Cork, Ireland.

[¥] Both authors contributed equally to this work.

* Corresponding author: Dr F. Jerry Reen, School of Microbiology, University College Cork, Cork, Ireland. E-mail j.reen@ucc.ie; Phone +353 21 4901330.

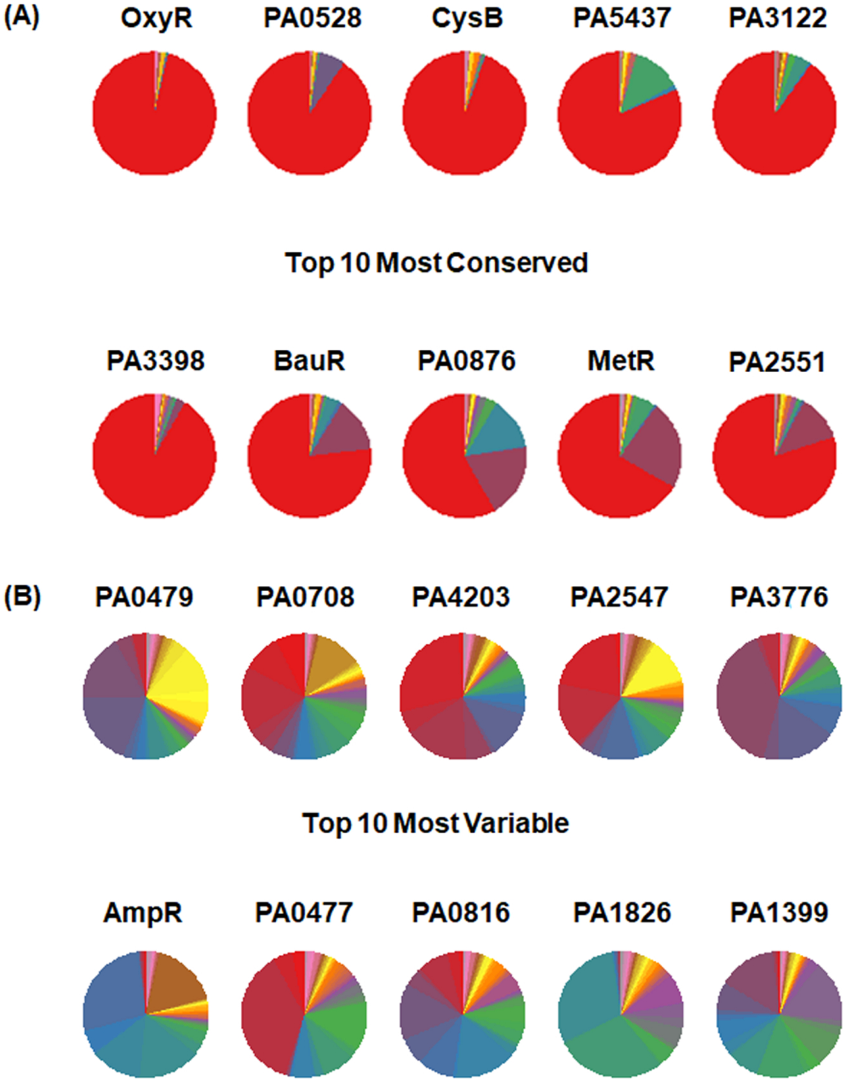

Supplementary File S3

Topology and Genomic Position of PAO1 LTTR Encoding Genes

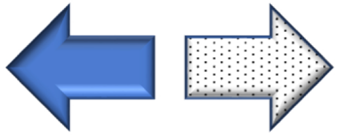

PA0056, PA0181, PA0191, PA0218, PA0477, PA0479, PA0491, PA0528, PA0708*, PA0739, PA0784, PA0816, PA0877, PA1003*, PA1184, PA1201, PA1223, PA1264, PA1309, PA1312, PA1328, PA1399, PA1738, PA1826, PA1859, PA1961, PA1998, PA2121, *PA2258, PA2267, PA2334, PA2417, PA2447, PA2469, PA2497, PA2534, PA2601, PA2877, PA2879, PA2921, PA3124, PA3135, PA3565, PA4109, PA4363, PA4989, PA5029, PA5218, PA5293, PA5382, PA5428,*

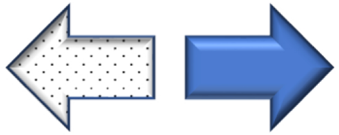

PA0032, PA0037, PA0133, PA0207, PA0448, PA0815, PA0876, PA1067, PA1128, PA1138, PA1145, PA1413, PA1422, PA1570, PA1853, PA2056, PA2076, PA2115, *PA2220, PA2316, PA2432, PA2492*, PA2510, PA2547, PA2551, PA2758, PA2834, PA2846, PA2848, PA2930, PA3122, PA3225, PA3321, PA3398, PA3433, PA3587, PA3594, PA3630, PA3711, PA3845, PA3776, PA3895, PA3995, PA4174, PA4203, PA4902, PA4914, PA5085, PA5179, PA5189, PA5344, PA5437*

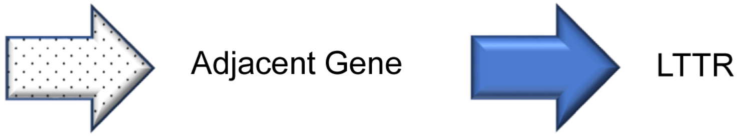

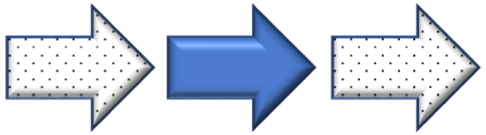

PA0152, PA0159, PA0233, PA0289, PA0701, PA2123, *PA2681, PA2838, PA3778, PA4145*

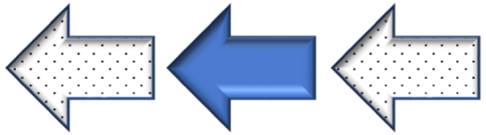

PA1141, PA2206, *PA2383*

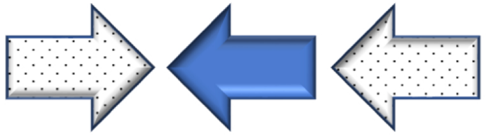

PA0123, PA0217, PA0272

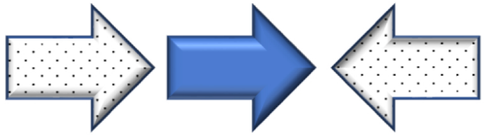

PA1754

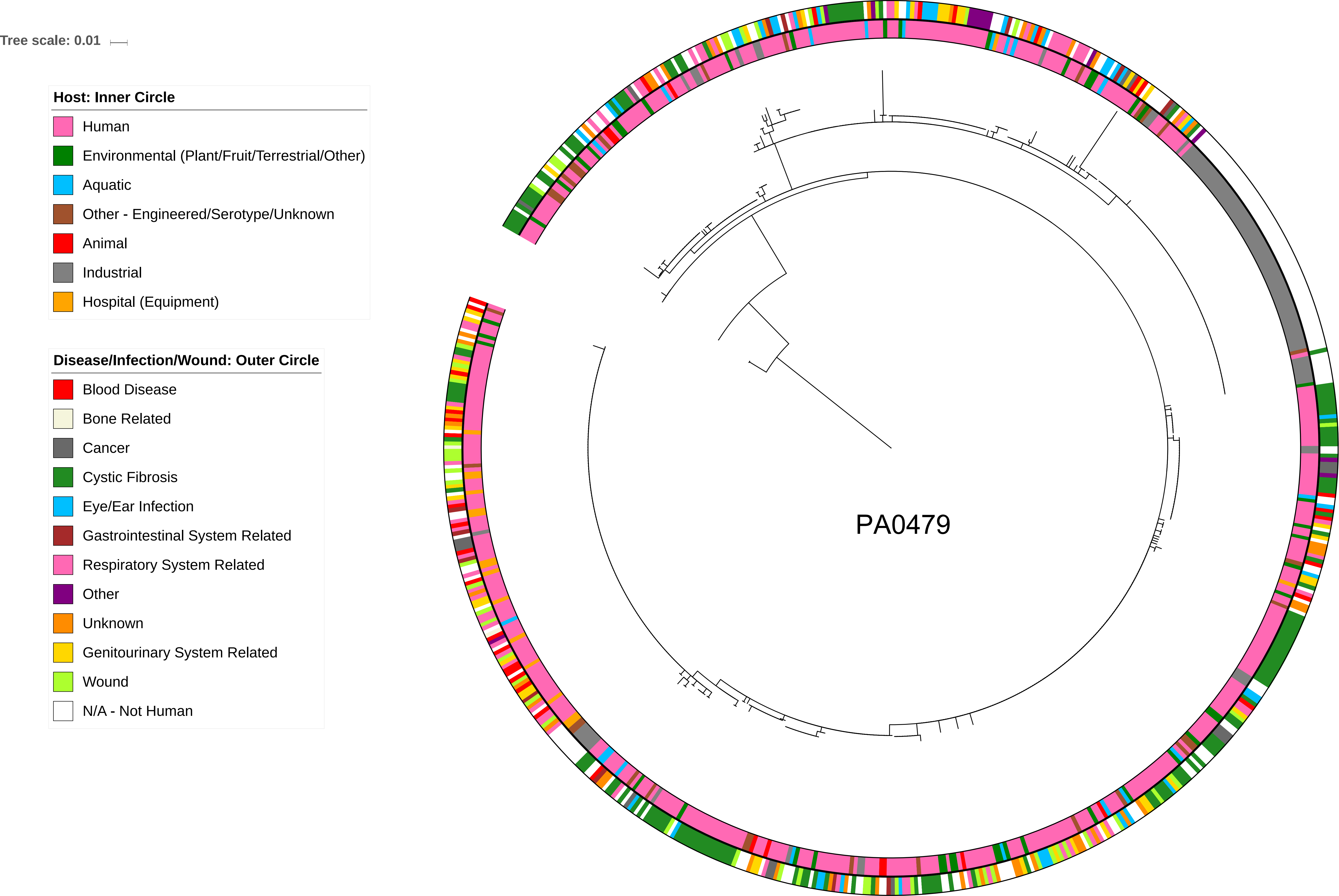
Tree scale: 0.01
Host: Inner Circle
Human
Environmental (Plant/Fruit/Terrestrial/Other)
Aquatic
Other - Engineered/Serotype/Unknown
Animal
Industrial
Hospital (Equipment)
Disease/Infection/Wound: Outer Circle
Blood Disease
Bone Related
Cancer
Cystic Fibrosis
Eye/Ear Infection
Gastrointestinal System Related
Respiratory System Related
Other
Unknown
Genitourinary System Related
Wound
N/A - Not Human
PA0479

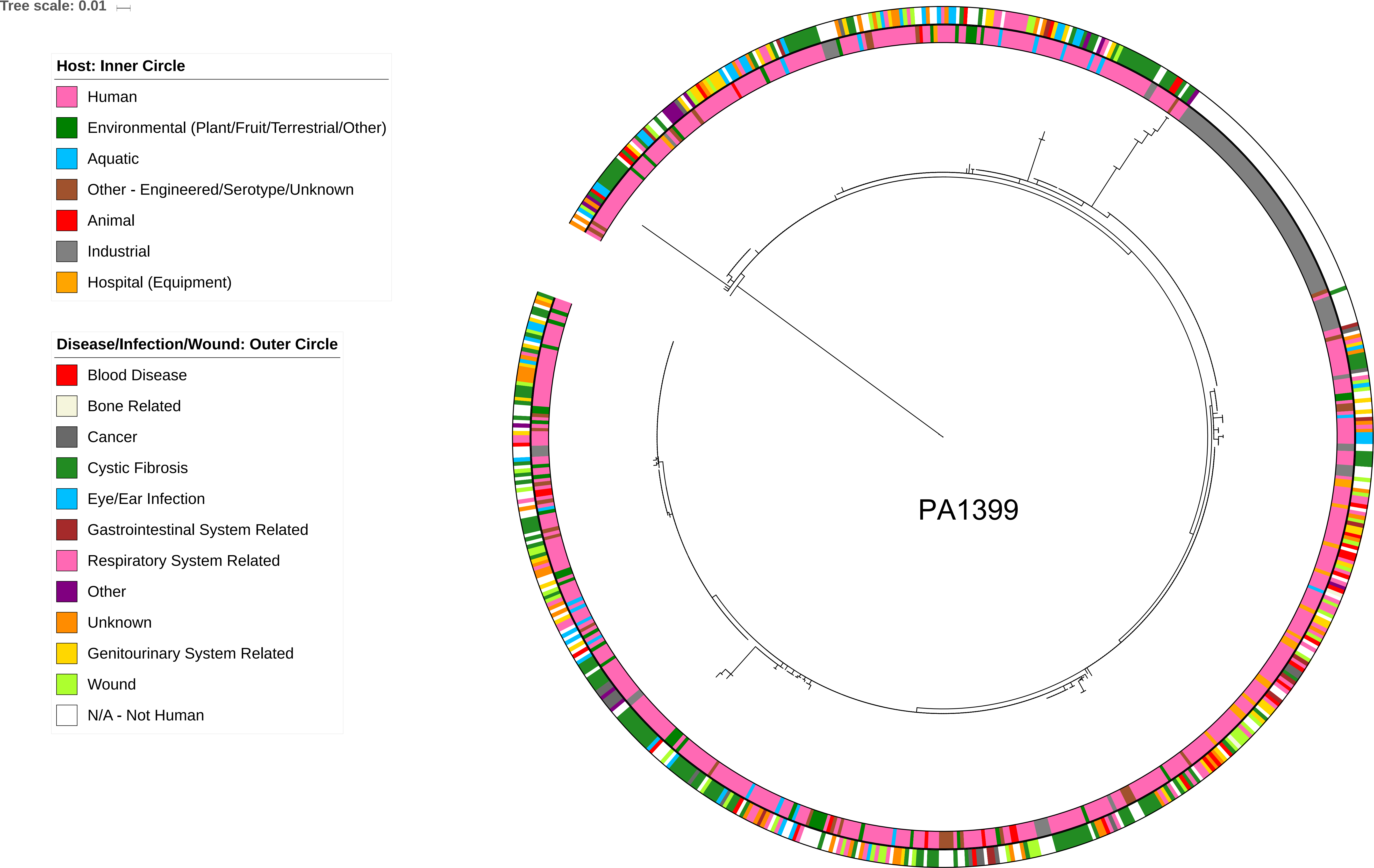
Tree scale: 0.01
Host: Inner Circle
Human
Environmental (Plant/Fruit/Terrestrial/Other)
Aquatic
Other - Engineered/Serotype/Unknown
Animal
Industrial
Hospital (Equipment)
Disease/Infection/Wound: Outer Circle
Blood Disease
Bone Related
Cancer
Cystic Fibrosis
Eye/Ear Infection
Gastrointestinal System Related
Respiratory System Related
Other
Unknown
Genitourinary System Related
Wound
N/A - Not Human
PA1399

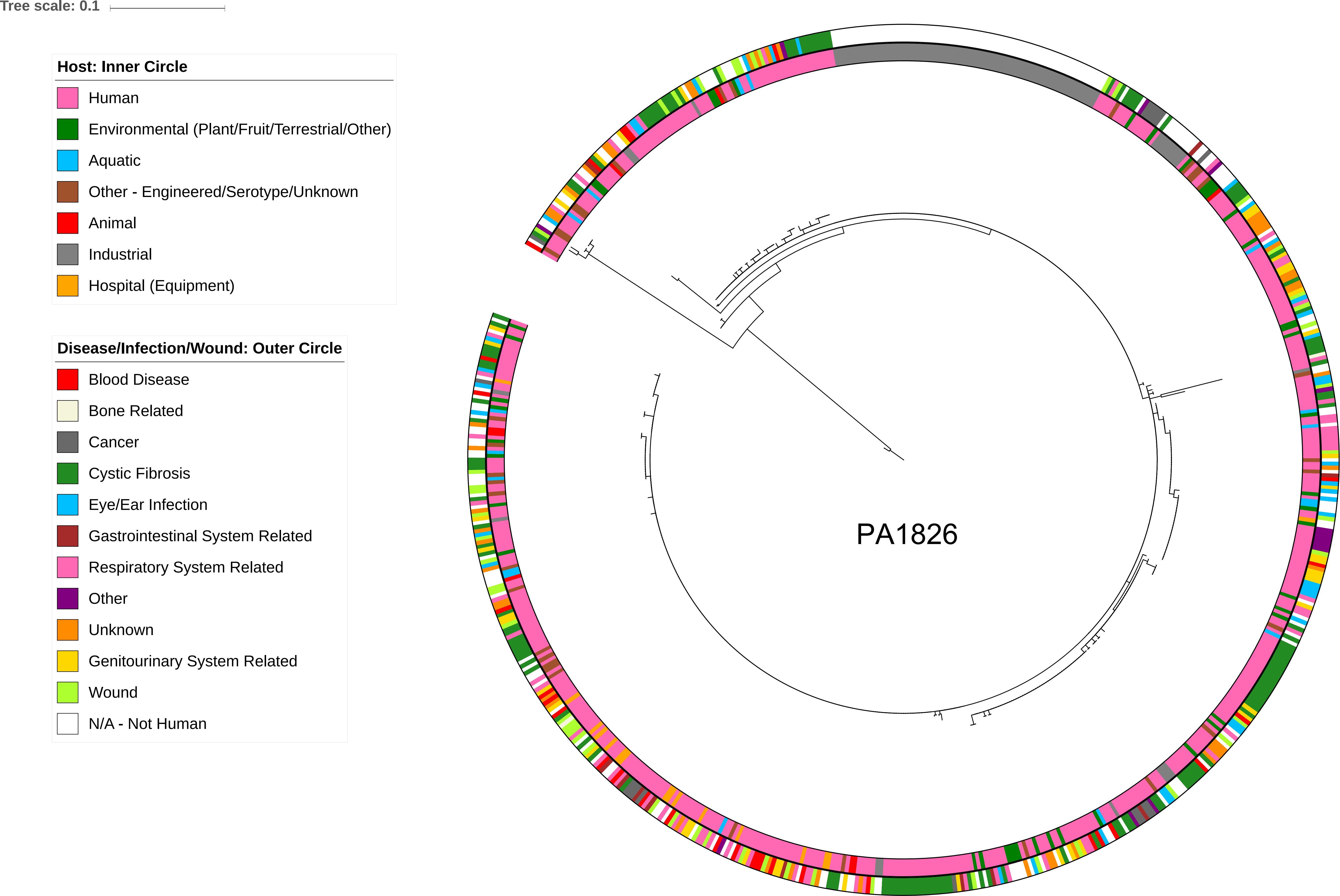
Tree scale: 0.1
Host: Inner Circle
Human
Environmental (Plant/Fruit/Terrestrial/Other)
Aquatic
Other - Engineered/Serotype/Unknown
Animal
Industrial
Hospital (Equipment)
Disease/Infection/Wound: Outer Circle
Blood Disease
Bone Related
Cancer
Cystic Fibrosis
Eye/Ear Infection
Gastrointestinal System Related
Respiratory System Related
Other
Unknown
Genitourinary System Related
Wound
N/A - Not Human
PA1826

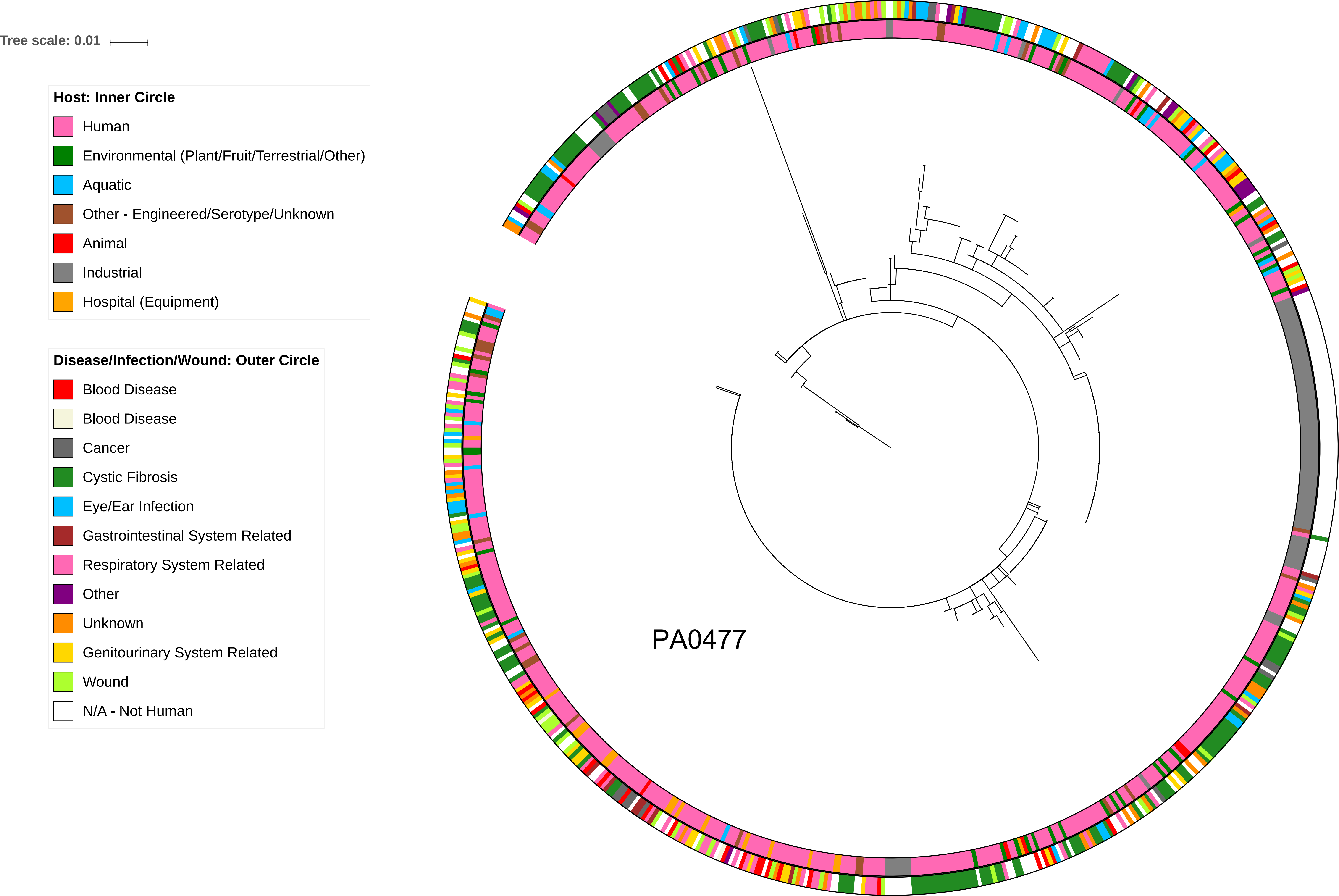
Tree scale: 0.01
Host: Inner Circle
Human
Environmental (Plant/Fruit/Terrestrial/Other)
Aquatic
Other - Engineered/Serotype/Unknown
Animal
Industrial
Hospital (Equipment)
Disease/Infection/Wound: Outer Circle
Blood Disease
Blood Disease
Cancer
Cystic Fibrosis
Eye/Ear Infection
Gastrointestinal System Related
Respiratory System Related
Other
Unknown
Genitourinary System Related
Wound
N/A - Not Human
PA0477

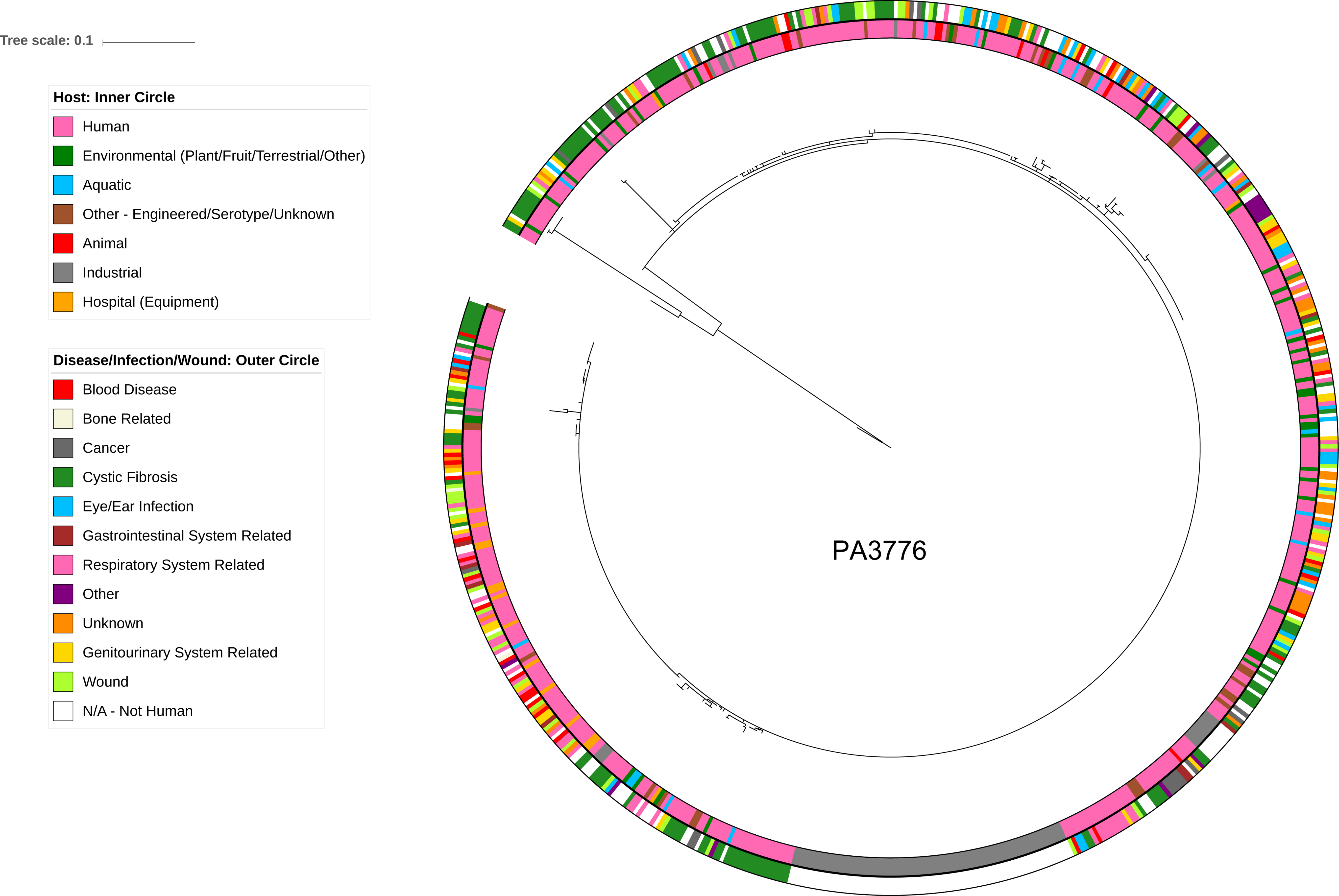

Tree scale: 0.1
Host: Inner Circle
Human
Environmental (Plant/Fruit/Terrestrial/Other)
Aquatic
Other - Engineered/Serotype/Unknown
Animal
Industrial
Hospital (Equipment)
Disease/Infection/Wound: Outer Circle
Blood Disease
Bone Related
Cancer
Cystic Fibrosis
Eye/Ear Infection
Gastrointestinal System Related
Respiratory System Related
Other
Unknown
Genitourinary System Related
Wound
N/A - Not Human
PA3776

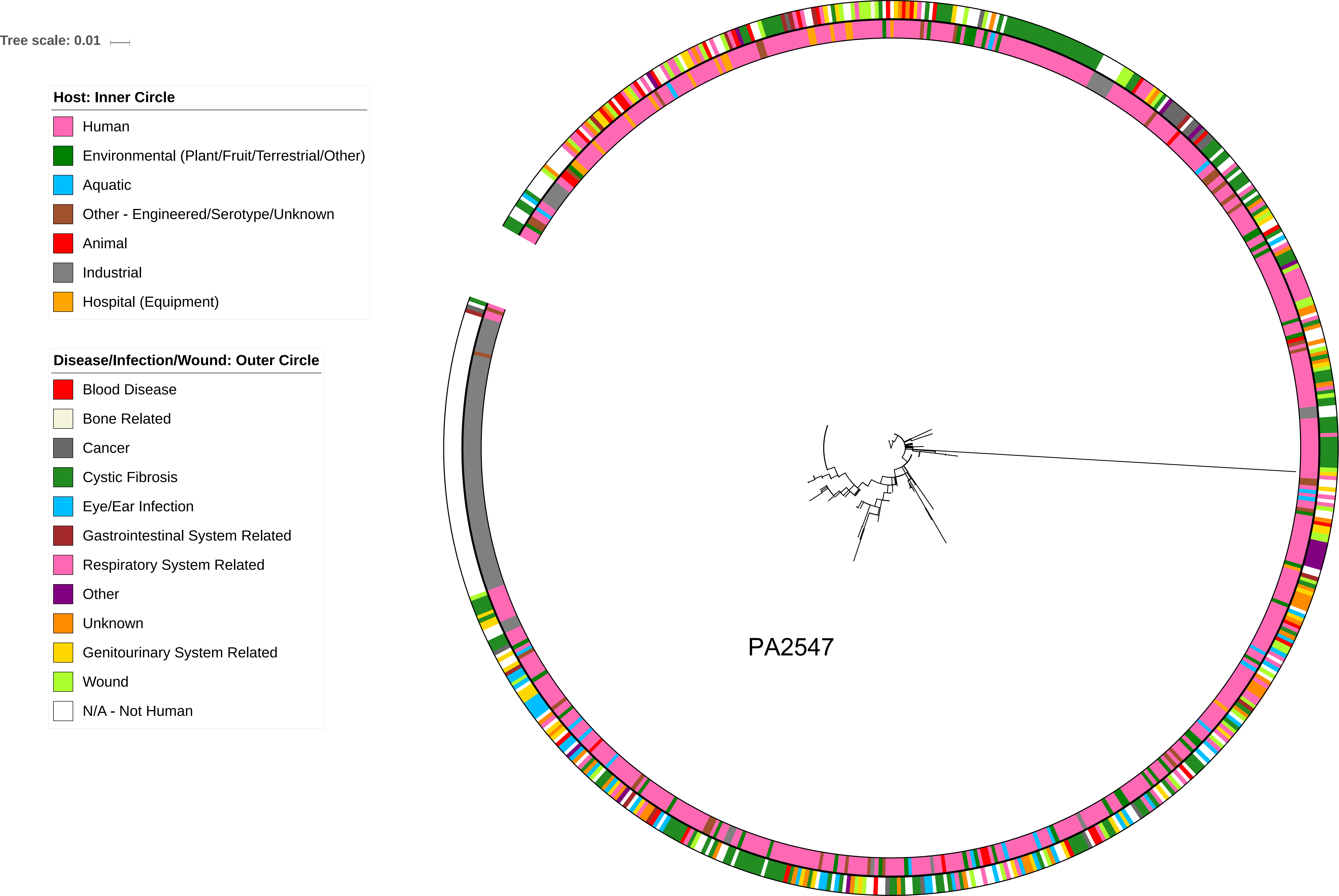
Tree scale: 0.01
Host: Inner Circle
Human
Environmental (Plant/Fruit/Terrestrial/Other)
Aquatic
Other - Engineered/Serotype/Unknown
Animal
Industrial
Hospital (Equipment)
Disease/Infection/Wound: Outer Circle
Blood Disease
Bone Related
Cancer
Cystic Fibrosis
Eye/Ear Infection
Gastrointestinal System Related
Respiratory System Related
Other
Unknown
Genitourinary System Related
Wound
N/A - Not Human
PA2547

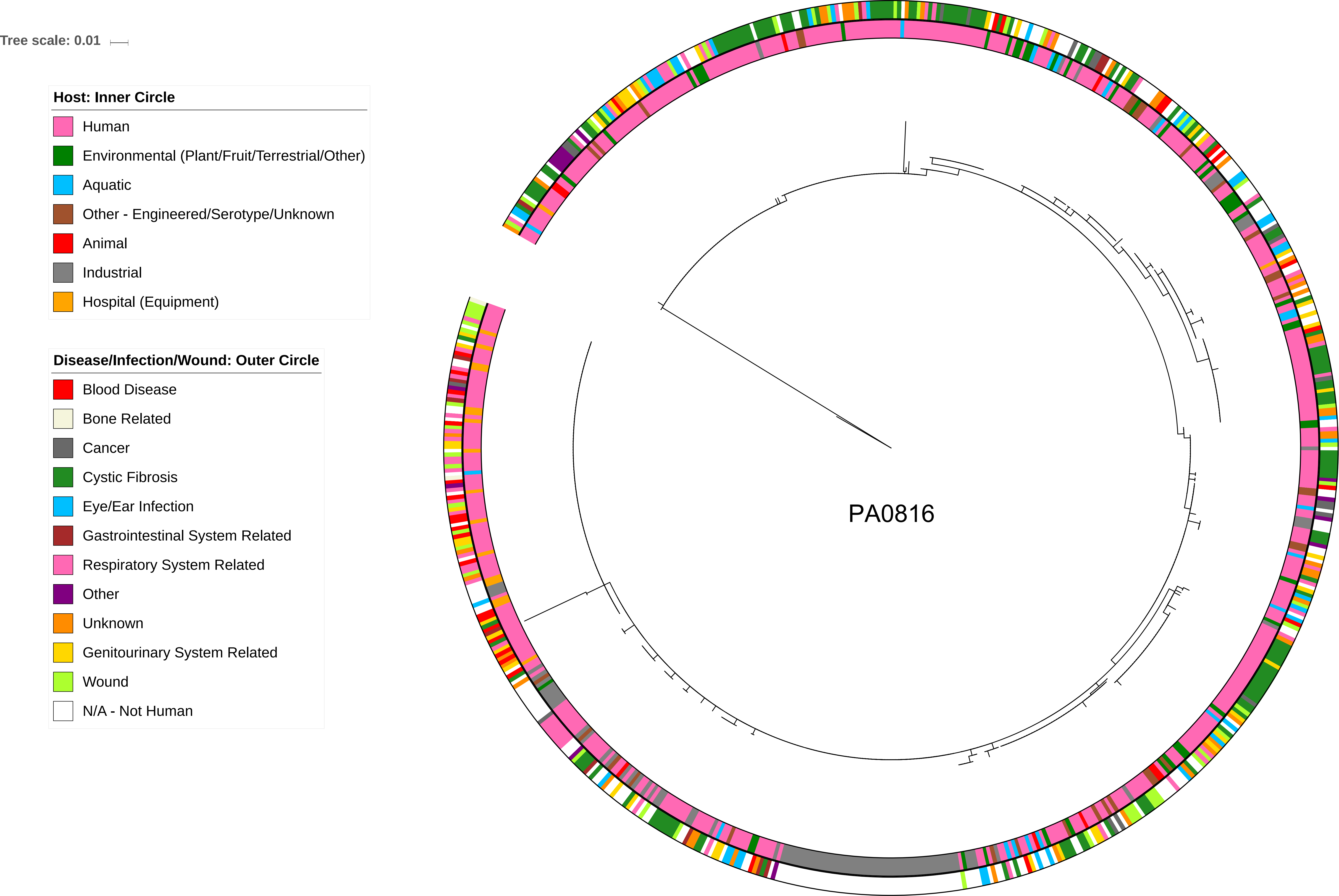
Tree scale: 0.01
Host: Inner Circle
Human
Environmental (Plant/Fruit/Terrestrial/Other)
Aquatic
Other - Engineered/Serotype/Unknown
Animal
Industrial
Hospital (Equipment)
Disease/Infection/Wound: Outer Circle
Blood Disease
Bone Related
Cancer
Cystic Fibrosis
Eye/Ear Infection
Gastrointestinal System Related
Respiratory System Related
Other
Unknown
Genitourinary System Related
Wound
N/A - Not Human
PA0816

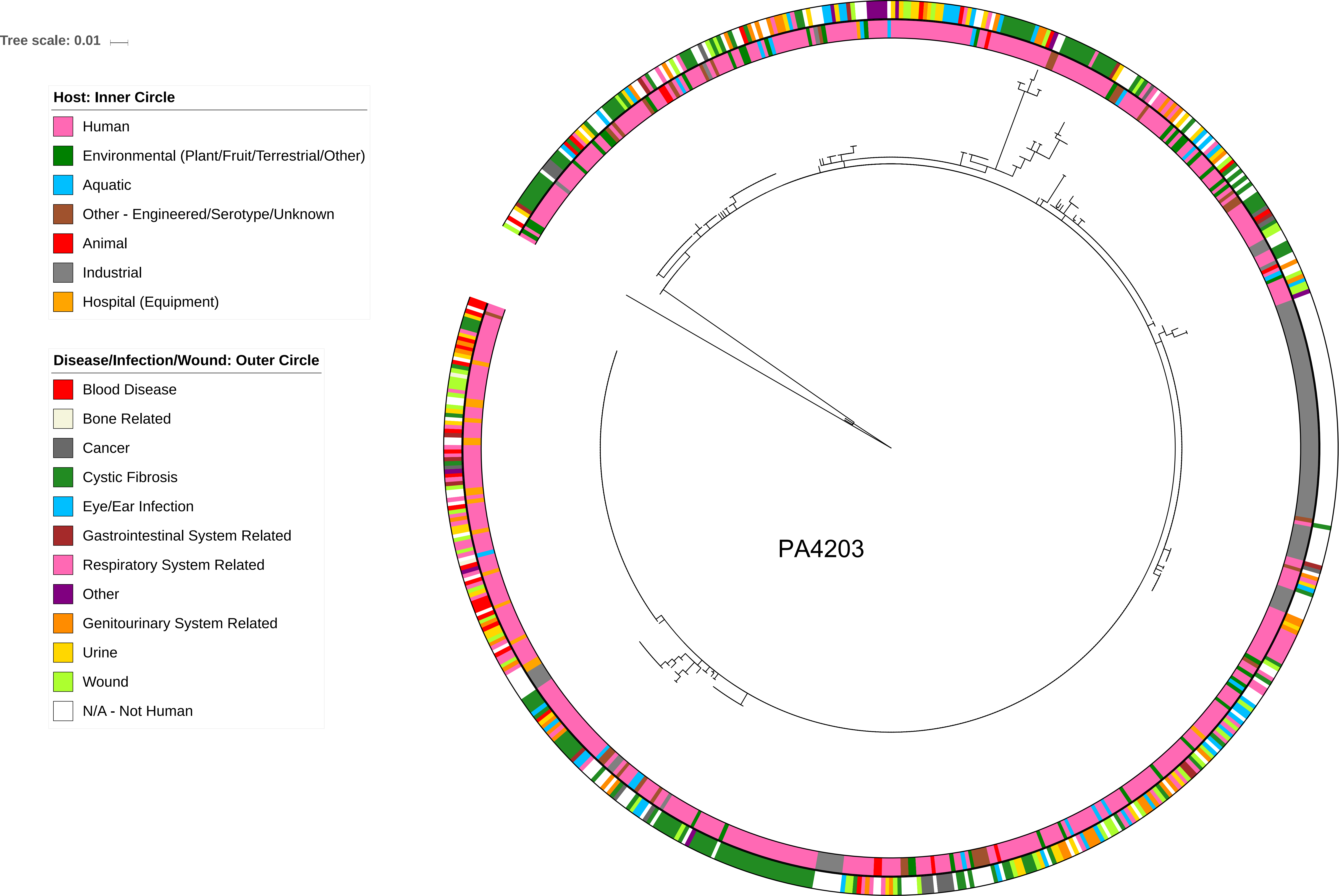
Tree scale: 0.01
Host: Inner Circle
Human
Environmental (Plant/Fruit/Terrestrial/Other)
Aquatic
Other - Engineered/Serotype/Unknown
Animal
Industrial
Hospital (Equipment)
Disease/Infection/Wound: Outer Circle
Blood Disease
Bone Related
Cancer
Cystic Fibrosis
Eye/Ear Infection
Gastrointestinal System Related
Respiratory System Related
Other
Genitourinary System Related
Urine
Wound
N/A - Not Human
PA4203

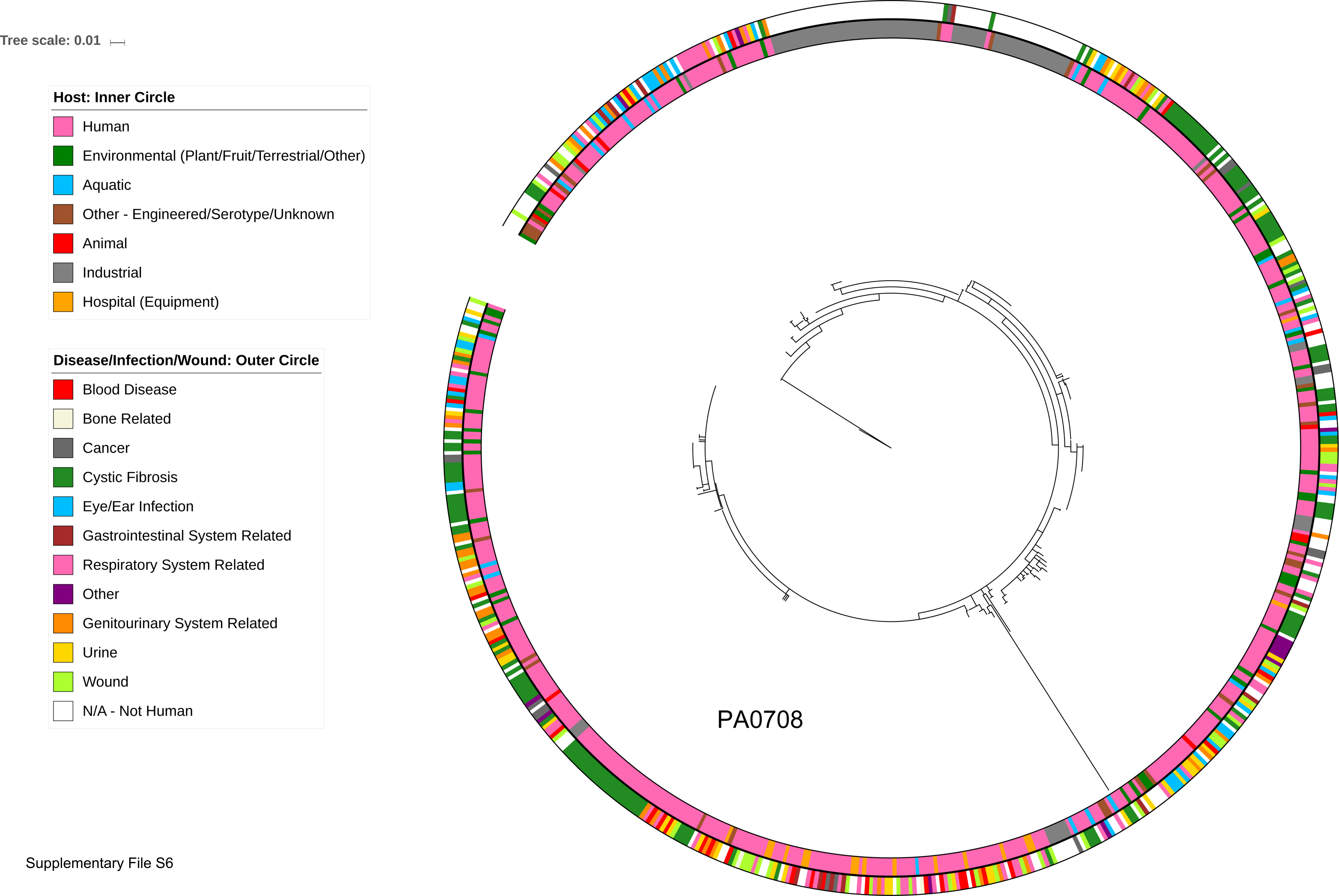

Supplementary File S6

Topology and Genomic Position of PA14 LTTR Encoding Genes

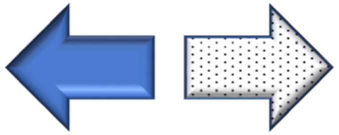

PA14_00680, PA14_02290, PA14_02390, PA14_02660, PA14_06240, PA14_06260, PA14_06400, PA14_06880, PA14_09570, PA14_09910, PA14_10120, PA14_10830, PA14_12140, PA14_13510, PA14_14280, PA14_15210, PA14_16380, PA14_17380, PA14_17790, PA14_17900, PA14_19670, PA14_20130, PA14_21080, PA14_22470, PA14_23730, PA14_26150, PA14_27250, PA14_27280, PA14_27440, PA14_28420, PA14_31560, PA14_31630, PA14_32200, PA14_32410, PA14_33170, PA14_34690, *PA14_37220, PA14_37660, PA14_37910, PA14_37940, PA14_44180, PA14_46060, PA14_46170, PA14_49590, PA14_49680, PA14_49790, PA14_50600, PA14_52930, PA14_53730, PA14_56740, PA14_65970, PA14_66490, PA14_68920, PA14_69880, PA14_71090, PA14_71640,*

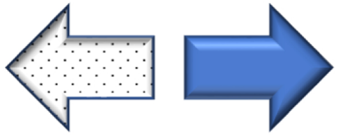

PA14_00400, PA14_00460, PA14_01640, PA14_05850, PA14_10090, PA14_10800, PA14_18200, PA14_22550, PA14_23540, PA14_23700, PA14_26270, PA14_26860, PA14_26880, PA14_30450, PA14_31780, PA14_32360, PA14_32700, PA14_32970, PA14_33440, PA14_35250, PA14_35380, *PA14_37140, PA14_38680, PA14_39160, PA14_40440, PA14_40550, PA14_40910, PA14_42060, PA14_46330, PA14_47080, PA14_47270, PA14_47310, PA14_47880, PA14_48500, PA14_48770, PA14_49110, PA14_51340*, PA14_52920, PA14_53720, PA14_54130, PA14_54710, PA14_55150, PA14_64780, PA14_64910, PA14_67170, PA14_68420, PA14_68550, PA14_70560, PA14_71750*

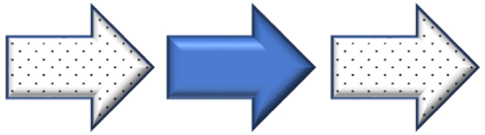

PA14_01890, PA14_01980, PA14_02870, PA14_03780, PA14_33840, *PA14_36180, PA14_49640*

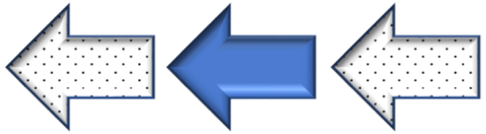

PA14_15210, PA14_27400, PA14_29440, PA14_30970, *PA14_37120, PA14_55250,*

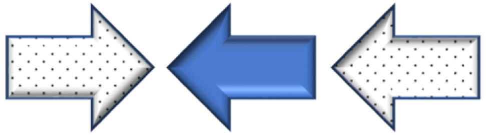

PA14_01500, PA14_02650, PA14_03530, PA14_10320, *PA14_41870,*

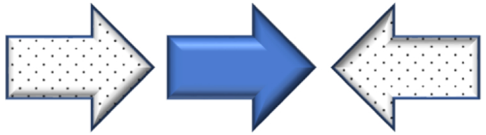

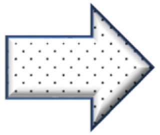

Adjacent Gene

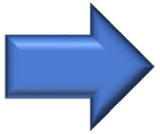

LTTR

Supplementary File S7

Supplementary File S9. Comparison of ten most and least conserved PA14 LTTR proteins analysed in 2021 and 2023*.

Gene ID	Locus	Sn	Vn	V	Gene ID	Locus	Sn	Vn	V
**Least variable, most conserved**					**Most variable, least conserved**				
PA14_70560	OxyR	2224 (8027)	16 (60)	0.72 (0.75)	PA14_30970		1457 (5279)	136 (292)	9.33 (5.53)
PA14_01640	BauR	2214 (7977)	24 (75)	1.08 (0.94)	PA14_54710	PA0191	2206 (7919)	136 (255)	6.17 (3.22)
PA14_71640	PA5428	2223 (8020)	25 (75)	1.13 (0.94)	PA14_06260	PA0479	2174 (7849)	126 (273)	5.80 (3.47)
PA14_02650	PA0217	2223 (7997)	25 (73)	1.13 (0.91)	PA14_10090		363 (1096)	20 (36)	5.51 (3.30)
PA14_71750	PA5437	2221 (8014)	25 (69)	1.13 (0.86)	PA14_37940	CynR	2174 (7819)	118 (211)	5.43 (2.70)
PA14_41870	CysB	2221 (8031)	26 (83)	1.17 (1.03)	PA14_54130	PA0784	2214 (7962)	111 (191)	5.01 (2.40)
PA14_02660	PA0218	2217 (7994)	26 (90)	1.17 (1.13)	PA14_29440	PA2681	2175 (7860)	106 (234)	4.87 (2.99)
PA14_46060	GbuR	2208 (7959)	27 (78)	1.22 (0.98)	PA14_09570	NmoR	2160 (7731)	105 (236)	4.86 (3.05)
PA14_23730	PA3122	2223 (8002)	28 (90)	1.26 (1.13)	PA14_46330	PA1399	2194 (7901)	99 (212)	4.51 (2.68)
PA14_06880	PA0528	2222 (8029)	28 (70)	1.26 (0.87)	PA14_51340	MvfR	2174 (7822)	96 (232)	4.42 (2.97)

***2023 analysis is presented in parentheses**

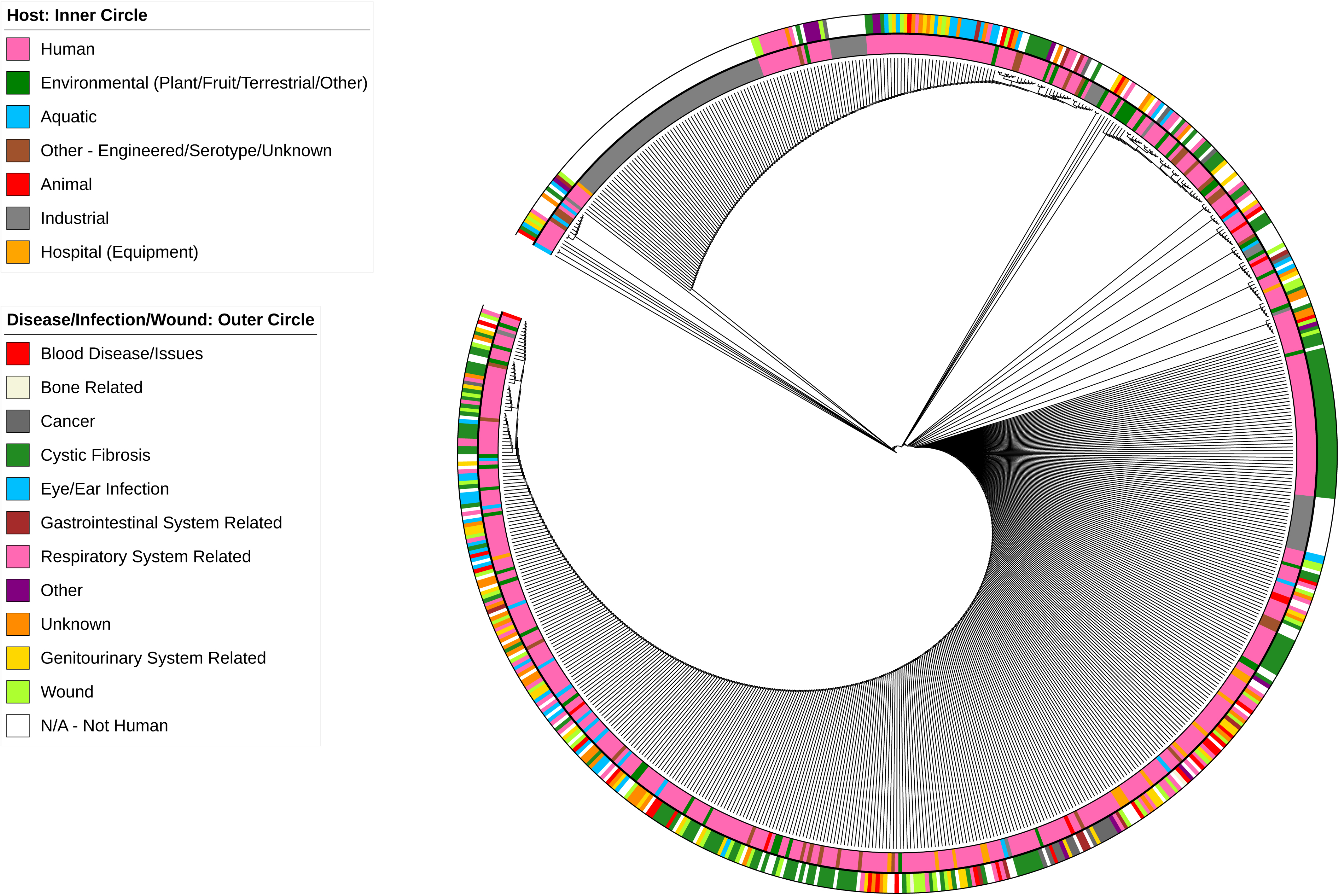

Supplementary File S10

pqsA Promoter MEME Analysis of LTTR motifs

(i)

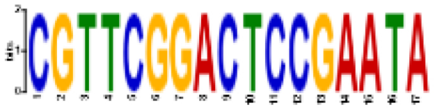

(ii)

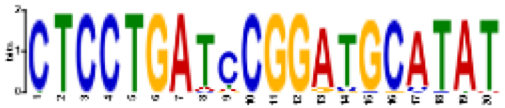

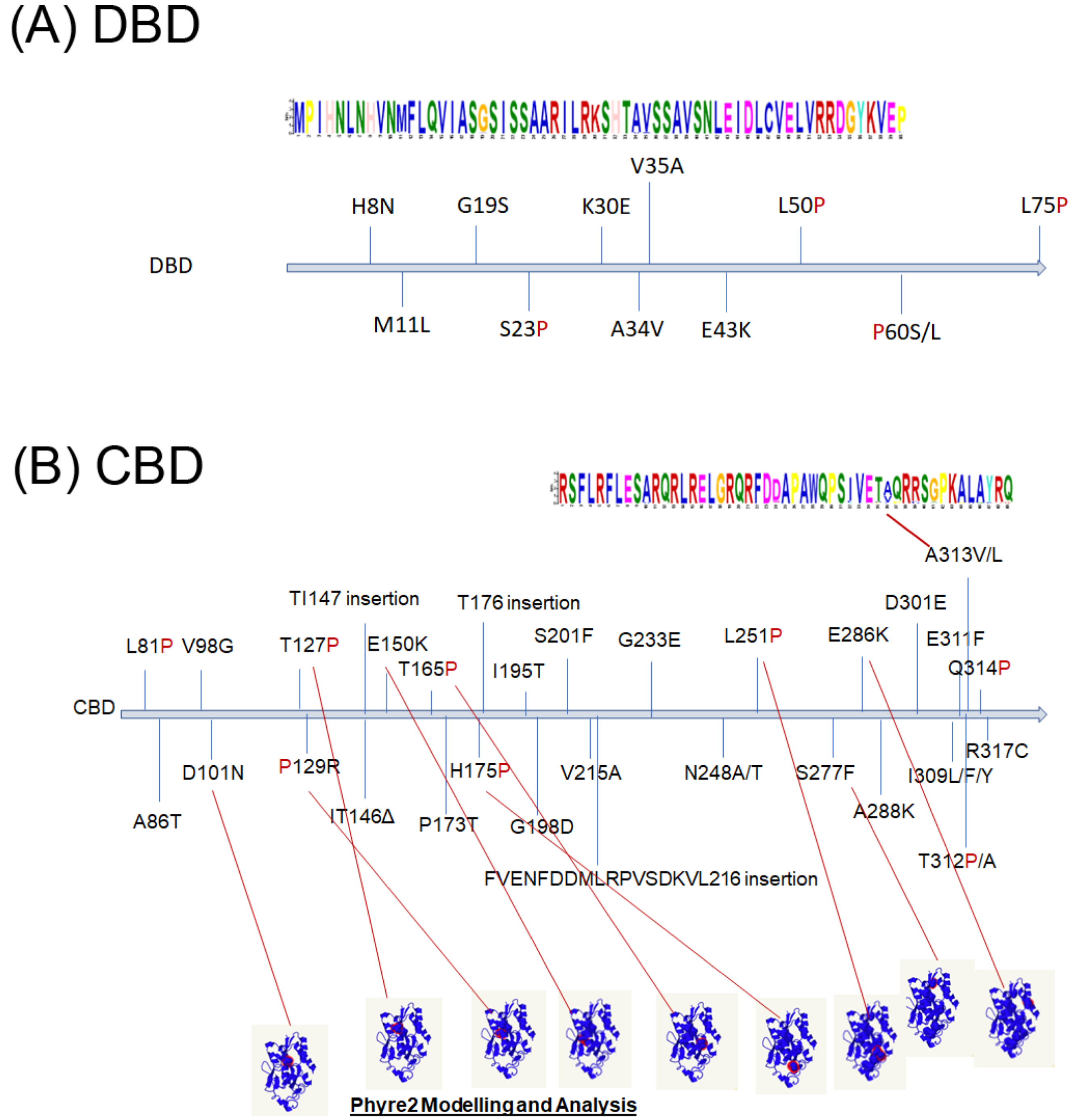

Supplementary File S12

	Ala	Cys	Asp	Glu	Phe	Gly	His	Ile	Lys	Leu	Met	Asn	Pro	Gln	Arg	Ser	Thr	Val	Trp	Tyr
**Ala**												6					1	1		
**Cys**						1									9					1
**Asp**						1														
**Glu**			10			1			**1**									2		
**Phe**																1				
**Gly**				1														2		
**His**										1					3					
**Ile**																				
**Lys**				4																
**Leu**					1		1	2					3			1				
**Met**																				
**Asn**	6		2					1												
**Pro**							3			6				9		1	2			
**Gln**																				
**Arg**							1			1			1							
**Ser**		1				4							1							
**Thr**	1							2					1							
**Val**	33																			
**Trp**															1					
**Tyr**		1	1																	

Supplementary File S13

Supplementary Table File S14. Table showing the representative homologue selected for further analysis from each of the different groups. Each of 7 strains represents a number of homologues present in PqsR.

**Identity Number**	**Strain**	**Number of homologues represented**	**Other amplified homologues represented**
1	CF175*	4	CF208*
2	PA01	467	BR177*<br>BR257*<br>CLONE C*<br>CF95*<br>X25409*<br>SG17M*<br>Rumen 8080 - 4
3	PA14	146	BR642*<br>Rumen 8080 - 1<br>Rumen 8080 - 2<br>Rumen 8043 - 3<br>Rumen 7987 - 19
4	Rumen 8080 - 3	7	Rumen 7987 - 18<br>Rumen 8131 - 2
5	7NSK2	1	N/A
6	CF194*	1	N/A
7	PA7	1	N/A

* Finnan S, Morrissey JP, O'Gara F, Boyd EF. Genome diversity of *Pseudomonas aeruginosa* isolates from cystic fibrosis patients and the hospital environment. *J Clin Microbiol.* 2004 42(12):5783-92. doi: 10.1128/JCM.42.12.5783-5792.2004.

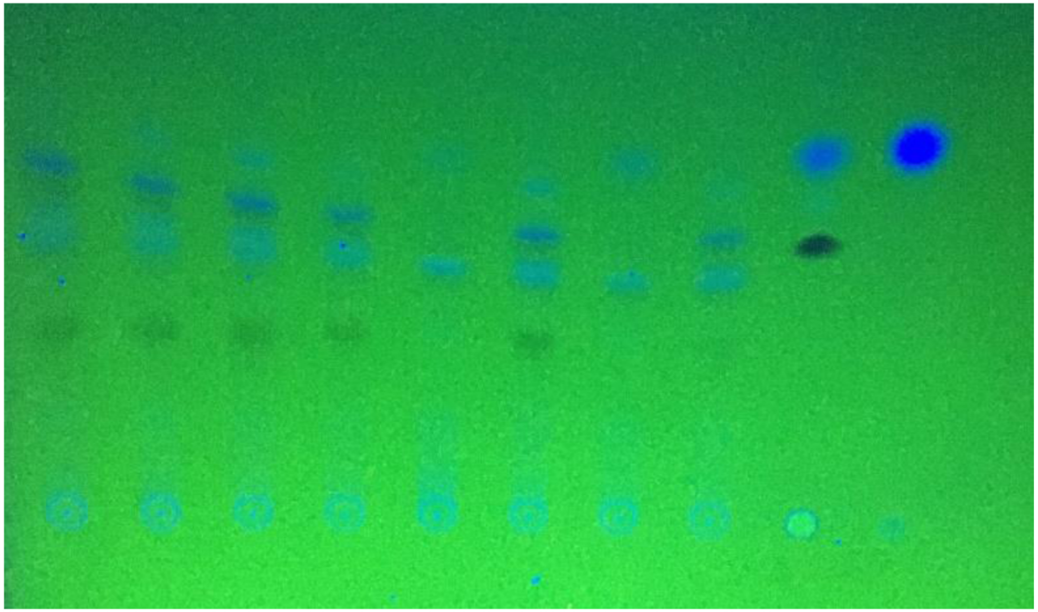

Cluster No: **1** **2** **3** **4** **5** **6** **PAO1 *pqsR*-** **PAO1** **PQS/ HHQ** **Anthranilic acid**

(i) HHQ (1 mM)

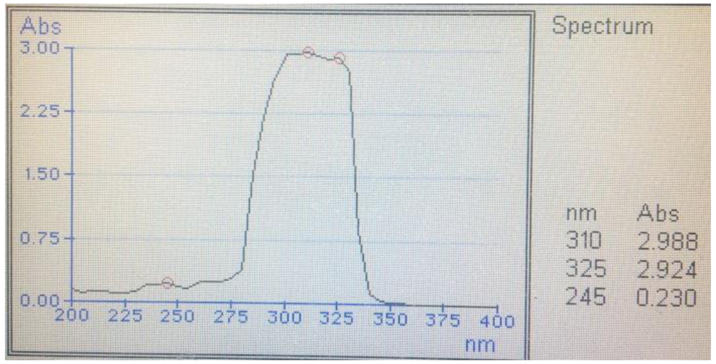

(ii) Cluster 1

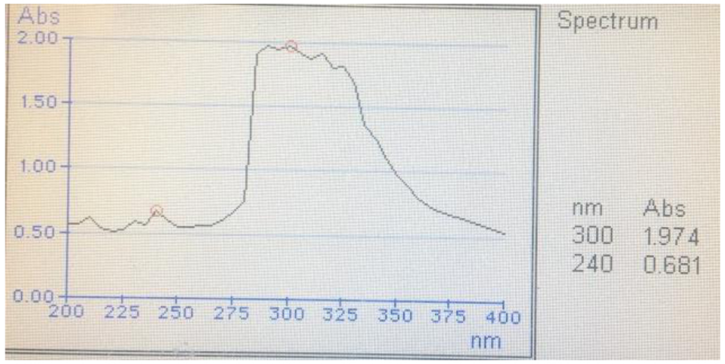

(iii) Cluster 2

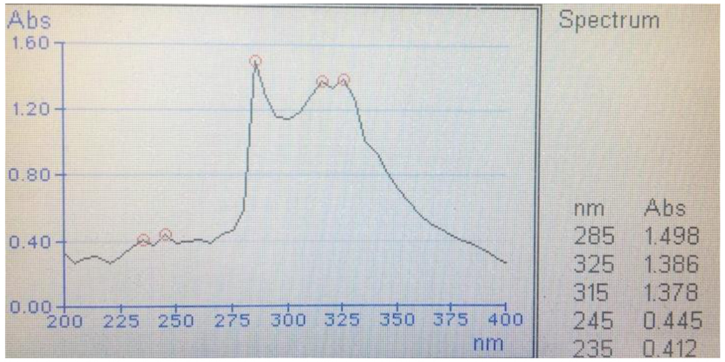

(iv) Cluster 3

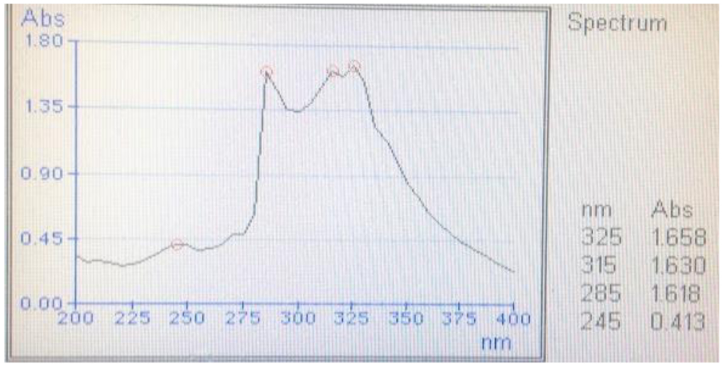

(v) Cluster 4

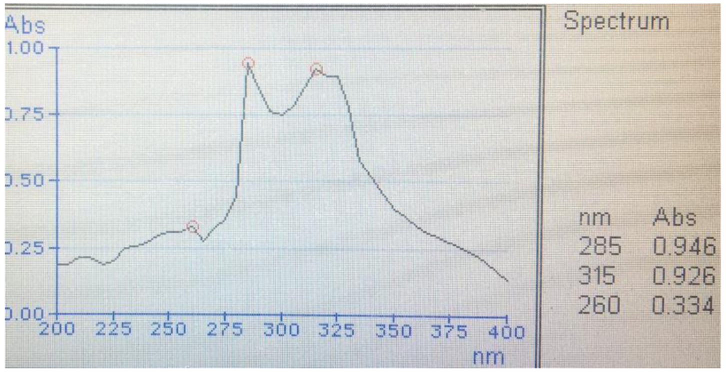

(vi) Cluster 5

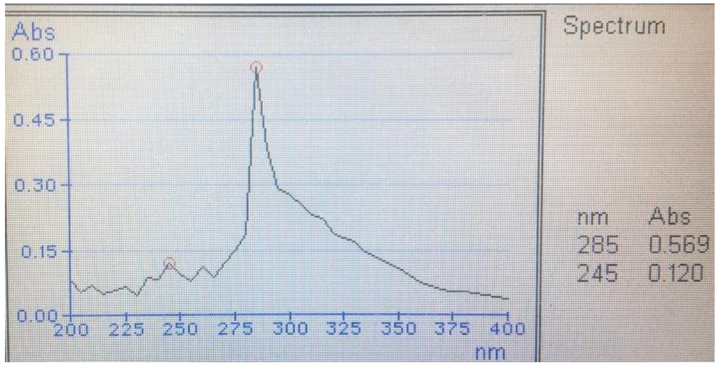

(vii) Cluster 6

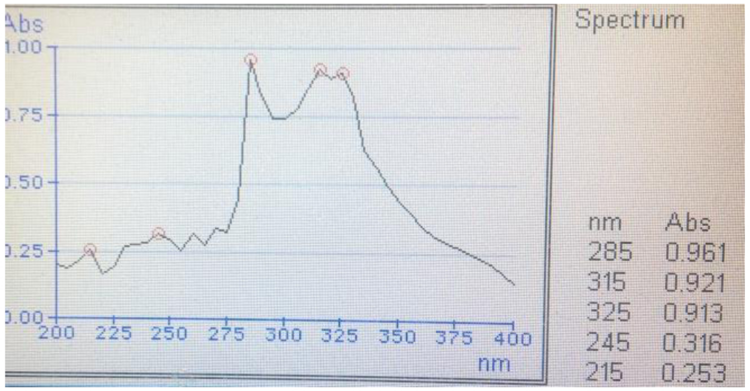

(viii) PAO1 *pqsR-*

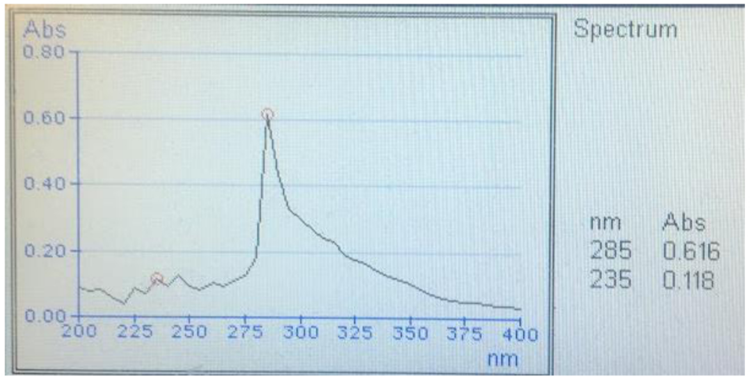

(ix) PAO1

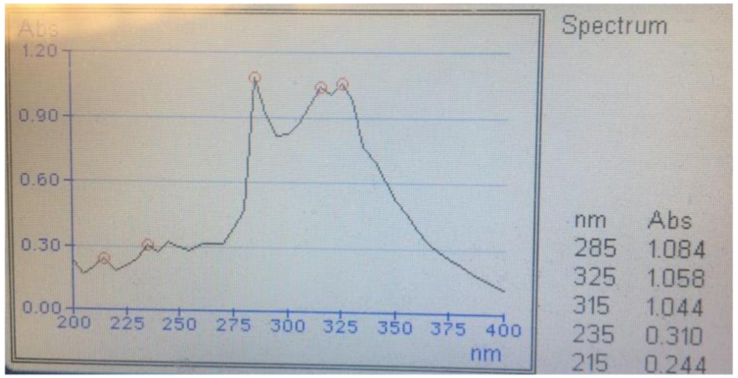

(x) PAO1 *pqsA-*

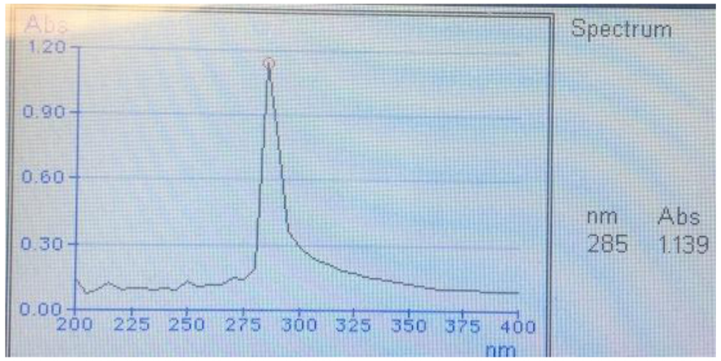

(xi) PQS (1 mM)

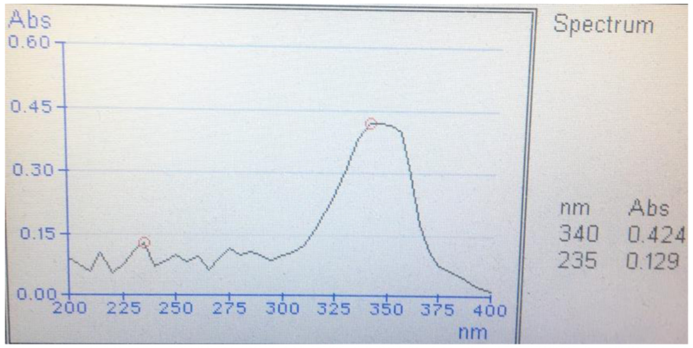

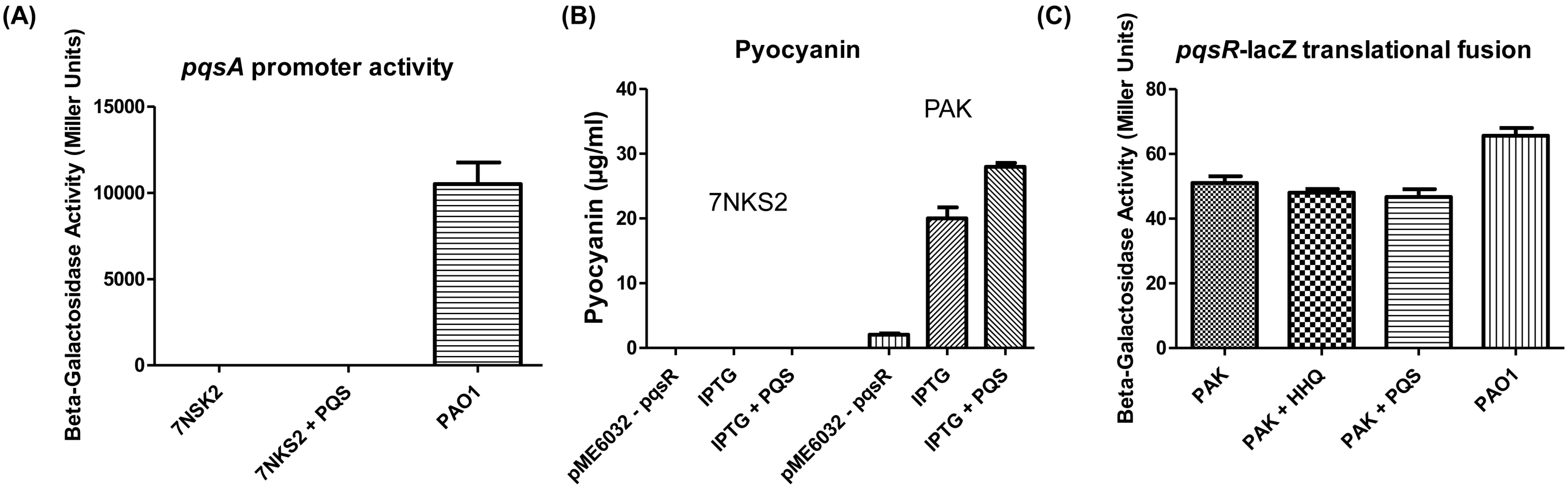
(A)
pqsA promoter activity
Beta-Galactosidase Activity (Miller Units)
15000
10000
5000
0
7NSK2
7NKS2 + PQS
PAO1
(B)
Pyocyanin
Pyocyanin (µg/ml)
40
30
20
10
0
PAK
7NKS2
pME6032 - pqsR
IPTG
IPTG + PQS
pME6032 - pqsR
IPTG
IPTG + PQS
(C)
pqsR-lacZ translational fusion
Beta-Galactosidase Activity (Miller Units)
80
60
40
20
0
PAK
PAK + HHQ
PAK + PQS
PAO1

Supplementary File Legends

Supplementary File S3. Pie Chart visualisation of the top 10 most conserved and top 10 most variable core LTTRs. Image created using ggplot.

Supplementary File S5. Topological arrangements of LTTR proteins with the repertoires of genes adopting those configurations presented on the right side of each arrangement. LTTR encoding gene is highlighted in blue, while the adjacent genes (not necessarily the transcriptional target) are white patterned. Gene numbers refer to the PAO1 annotation, with those in bold being encoded between the origin and terminus. Green highlighted genes denote those of the 10 most conserved (low variance), while red highlighted genes denote the 10 least conserved (high variance) LTTR encoding genes.

Supplementary File S6. Metadata cluster trees of 9 of the top 10 most variable LTTR proteins.

Supplementary File S7. Topology and Genomic Position of PA14 LTTR Encoding Genes

Supplementary File S9. Comparison of ten most and least conserved PA14 LTTR proteins analysed in 2021 and 2023.

Supplementary File S10. PqsR metadata tree with branch lengths removed.

Supplementary File S11. Motif analysis of the *pqsA* promoter from the Cluster representative sequences suggests conservation of the putative PqsR binding motifs. MEME Analysis performed on sequences identified in Clustal Omega alignments

upstream of the *pqsA-E* operon is shown.

Supplementary File S12. SNP analysis of the PqsR protein variants represented in the IMG database. **(A)** DNA-Binding Domain variants and **(B)** Co-Inducer Binding Domain variants. MEME analysis of each region capturing the diversity across each of the 49 variants is presented above each domain. The position of the SNP is presented in red on a Phyre2 model for the CDB.

Supplementary File S13. SNP Analysis of PqsR variance rooted using the PA14 sequence. Numbers in each section note the frequency of SNPs related to each amino acid change across the entire PqsR gene. Tows represent the amino acid position in the PA14 strain, while columns represent transitions observed in the variants encoded in the *P. aeruginosa* genomes e.g. Glu-Ala occurs 10 times.

Supplementary File S14. Table showing the representative homologue selected for further analysis from each of the different groups.

Supplementary File S15. Thin Layer Chromatography showing separation of extracted PQS (Rf value ~0.67) and HHQ (Rf value ~0.41) following complementation of PAO1 *pqsR*-with pBBR1-MCS5*pqsR* representative variants. Spots (left to right); Cluster 1-6 (30 µl each), PAO1 *pqsR*$^{-}$ (30 µl), PQS (upper spot – 2 µl of 10 mM solution), HHQ (lower spot - 2 µl of 10 mM solution) and Anthranilic acid (2 µl of 10 mM solution).

Supplementary File S16. Spectrophotometric analysis of HHQ production following complementation of PAO1 *pqsR*-with pBBR1-MCS5*pqsR* representative variants.

Supplementary File S17. Downstream analysis of PQS/PYO loss of function in the *P.*

aeruginosa 7NSK2 strain. (**A**) *pqsA*-lacZ promoter fusion analysis in 7NKS2 shows absence of activity, even in the presence of PQS signal, suggesting loss of function is not due to the biosynthetic genes. **(B)** PYO production is restored in PAK upon introduction of a functional PqsR, but not in 7NKS2, suggesting downstream factors are responsible. **(C)** *pqsR*-lacZ translational fusion analysis in PAK suggests that *pqsR* translation is not affected in the strain. Data presented is the average of three independent biological replicates.
